# Microbial dynamics and metabolic activity during fish decay under aerobic and anaerobic conditions: insights into microbial fossilization

**DOI:** 10.3389/fmicb.2026.1783163

**Published:** 2026-04-16

**Authors:** Maria A. Diaz-Mateus, Alex Smilovitis, Silvia J. Salgar-Chaparro, Kliti Grice

**Affiliations:** 1 Western Australian Organic & Isotope Geochemistry Centre, School of Earth and Planetary Sciences, Curtin University, Bentley, WA, Australia; 2 Curtin Corrosion Centre, WA School of Mines: Minerals, Energy and Chemical Engineering, Curtin University, Bentley, WA, Australia

**Keywords:** fish decay, fossilization process, microbial communities, microbial functional profiles, taphonomy

## Abstract

**Introduction:**

Understanding how environmental conditions influence early decay processes remains a central challenge in taphonomy, particularly regarding the relative roles of aerobic and anaerobic regimes. Because microorganisms drive soft-tissue decomposition, this study examines how contrasting oxygen conditions affect microbial community structure, metabolic potential, and the minerals formed during fish decay.

**Methods:**

Fish decay experiments were conducted under controlled aerobic and anaerobic conditions. O_2_, pH, and H_2_S were monitored using microprobe measurements, and water chemistry was characterized through major ion analyses. Microbial community composition was assessed using 16S rRNA gene sequencing, and from this, metabolic potential was predicted using PICRUSt2. SEM-EDS and XRD were used to identify the precipitate minerals.

**Results:**

Oxygen availability resulted in markedly distinct microbial assemblages and decay trajectories. Anaerobic conditions were associated with lower pH and elevated hydrogen sulfide concentrations, whereas aerobic conditions maintained higher pH. Predicted metabolic functions were broadly conserved between treatments. Aerobic decay was characterized by higher DIC, lower DOC, and enrichment of genes linked to complete organic matter oxidation, including β-oxidation pathways. In contrast, anaerobic decay exhibited higher DOC, lower DIC, and increased abundances of genes associated with fermentation and carbohydrate metabolism, consistent with incomplete degradation. Mineralogical analyses identified magnesium phosphates and sodium sulfates in both treatments.

**Discussion:**

Environmental oxygen availability strongly controls microbial metabolism and decay dynamics. Aerobic microbial communities promoted more extensive tissue decomposition, whereas anaerobic communities favored slower, fermentation-dominated decay. The early mineral phases identified in both treatments likely represent precursors to diagenetic minerals and provide new insights into mineralization pathways that may contribute to exceptional fossil preservation.

## 1 Introduction

Under typical environmental conditions, the decomposition of organic matter begins rapidly after an organism's death, mainly driven by cellular autolysis and microbial activity (Zhou and Byard, 2011; DeBruyn et al., 2025). In natural settings, carcasses are commonly exposed to scavenging and physical processes, such as transport by water or wind and erosion, which contribute to the fragmentation and dispersal of remains. Consequently, fossilization is considered a rare and complex phenomenon. Since the mid-20th century, researchers across multiple disciplines have employed diverse approaches to elucidate the mechanisms underlying fossil preservation. It is generally accepted that successful fossilization requires conditions that both inhibit decay and promote rapid mineral precipitation (Briggs and McMahon, 2016; Naimark et al., 2016). Nevertheless, the precise mechanisms of these processes remain incompletely understood.

Highly anaerobic environments, such as anoxic basins with fine-grained sediments, have traditionally been regarded as key settings for fossilization. These environments typically exhibit minimal bioturbation and scavenging, allowing carcasses to remain intact for extended periods (Allison and Briggs, 1993). Their reducing conditions limit autolysis, slowing the rate of decay (Raff et al., 2006). Moreover, burial within these sediments creates a highly localized microenvironment around the carcass, where ions released from decomposing tissues cannot easily diffuse. This concentration of solutes promotes authigenic mineral precipitation (Briggs and Kear, 1994), which is believed to facilitate the preservation of organic matter.

However, experimental taphonomy has not reached a consensus on whether aerobic or anaerobic conditions consistently lead to faster or slower decomposition. Some studies have reported that anoxic conditions are associated with reduced decay rates in various model organisms (Briggs and Kear, 1993a,b; Raff et al., 2008; Gostling et al., 2009), whereas others have found no significant difference between decay under anoxic and oxygenated conditions (Allison, 1988; Kidwell and Baumiller, 1990; Briggs and Kear, 1994; Mähler et al., 2023). Recent findings indicate that oxygen availability is not always the primary factor governing the preservation of organic matter. (Hancy and Antcliffe 2020) demonstrated that, in cnidarians, different tissues exhibit contrasting decay patterns: the hypostome decayed more rapidly under anaerobic conditions, whereas the actinopharynx decayed faster under aerobic conditions. These results suggest that tissue-specific responses, in conjunction with redox conditions, regulate the mechanisms and rates of soft-tissue degradation.

Furthermore, recent taphonomy studies have shown that additional environmental factors—such as sediment chemistry and water chemistry (including ion concentration, pH, and salinity), also play significant roles in modulating decay rates (Naimark et al., 2016; Newman et al., 2019; Gäb et al., 2020; Gibson et al., 2023). The complexities of organic matter degradation in natural environments are likely context-dependent rather than universal. Therefore, more controlled taphonomy studies are needed to advance our understanding of specific biostratinomic processes and environmental variables that influence decay.

Given the central role of microorganisms in the degradation of organic matter and the associated changes in water chemistry caused by microbial metabolism that can promote mineral precipitation (Jørgensen et al., 2019; Hoffmann et al., 2021)—microorganisms are believed to play a key role in the fossilization process. Controlled laboratory experiments using a range of model organisms have revealed two principal mechanisms by which microorganisms may facilitate the initial phases of fossil formation. First, microbial metabolic activity can drive the precipitation of isolated mineral phases, mineral crusts and, in some cases, complete mineralization of tissues of under different environmental conditions (Briggs and Kear, 1993c, 1994; Hof and Briggs, 1997; Sagemann et al., 1999; Mähler et al., 2020, 2022, 2023; Odin et al., 2024). Second, microorganisms have the potential to form pseudomorphs that replicate the morphology of the original organism, which are thought to act as structural templates for subsequent mineralization processes (Raff et al., 2008; Butler et al., 2015; Iniesto et al., 2016; Eagan et al., 2017).

To date, taphonomy experiments have begun incorporating molecular biology techniques to strengthen interdisciplinary efforts aimed at understanding the microbial mechanisms that drive decay and preservation. So far, 16S rRNA and ITS amplicon analyses have been used to identify the microorganisms associated with these processes (Mähler et al., 2022, 2023; Clements et al., 2025; Corthésy et al., 2024; Karačić et al., 2024). However, while determining the taxonomic composition of microbial communities allows for comparisons across time points or experimental conditions, it provides limited insight into their actual metabolic capabilities (Vital-Jácome et al., 2024). Taxonomic profiling can reveal which microorganisms are present and suggest their potential metabolic roles based on literature, but this approach overlooks the fact that microbial communities can exhibit functional redundancy, i.e., different taxa may perform similar functions (Chen et al., 2022). Consequently, changes in community composition over time may not necessarily reflect shifts in metabolic activity.

The present study aims to characterize changes in both bacterial community structure and metabolic function during the early stages of fish tissue decomposition under aerobic and anaerobic conditions, and to determine the extent to which these changes correlate with decay rates and mineral formation. These results will provide new insights into the specific microbiological mechanisms involved in degradation, mineralization, and, ultimately, the fossilization of organic matter. Collectively, the outcomes are expected to advance fundamental understanding of microbially mediated carbon cycling and mineral formation, and to establish methodological frameworks for applying molecular biology tools within experimental taphonomy.

## 2 Materials and methods

### 2.1 Material

Approximately 2 kg of fresh Longnose Emperor (*Lethrinus olivaceus*) filet, with skin and scales intact, was sourced from Hillseafoods (Perth, Western Australia). To maximize tissue freshness, collection was pre-arranged so that the fish could be retrieved immediately after gut removal. Specimens were transported to the laboratory in an insulated ice box with ice packs and processed within 2 h of purchase.

From the filet, 45 square tissue coupons (1.5 cm × 2 cm) were aseptically excised using a sterile scalpel. Each coupon retained a portion of muscle, skin, and scale tissue. Prior to incubation, all samples were rinsed with sterile deionized water to remove loosely attached debris.

### 2.2 Mesocosms

Mesocosms were prepared by mixing 1.5 kg of microbial mat material with 3 kg of pre washed white sand (Westbuild, WA, Australia). The microbial mat material was originally collected from the intertidal zone of Hamelin Pool, Western Australia, in April 2017 (Supplementary Figure S1) and subsequently maintained in Winogradsky columns at the Western Australian Organic and Isotope Geochemistry Centre (WA OIGC), Curtin University. The sand is a commercially sourced, quartz dominated construction sand that was autoclaved to use to sterilize it and prevent the introduction of exogenous microorganisms into the experimental setup. The mat–sand mixture (with a quantitative ratio of 1:2 mat: sand by mass) was thoroughly homogenized and evenly distributed among eight squared plastic containers, which had been sterilized by exposure to UV light for 1 h prior to use.

Microbial mats from Shark Bay were used as inoculum in this study due to the high microbial diversity and wide range of metabolic functions. More importantly, metabolic processes within these mats drive coupled reactions with environmental chemical species, often yielding mineral precipitates as end products (Visscher and Stolz, 2005; Suosaari et al., 2022). In addition, these mats provide a useful model system for studying fish decay dynamics under hypersaline conditions. Such settings are commonly associated with exceptional fossil preservation in the geological record (Heimhofer et al., 2010; Gallois et al., 2018; Dias and Carvalho, 2022), likely because hypersaline conditions enhance the stabilization of soft tissues relative to normal marine salinities (Gäb et al., 2020).

### 2.3 Experimental design

The experiment was designed to assess microbial dynamics during fish tissue decay under both aerobic and anaerobic conditions over a 16-day period. Four mesocosms were incubated under anaerobic conditions inside a Coy Laboratory Products Vinyl Anaerobic Chamber (Type B) maintained at 21 °C, under a stable atmosphere of 3–5 ppm O_2_. Four additional mesocosms were incubated under aerobic conditions in a fume hood at a constant temperature of 21 °C. Control mesocosms without fish tissue were also incubated under both aerobic and anaerobic conditions.

In each mesocosm, five fish tissue coupons were placed at the base of the plastic container and completely buried in mat-inoculated sand to a depth of approximately 1.5 cm. Containers were then filled with filter-sterilized seawater (collected from Hamelin Pool, Western Australia) to a level approximately 3 cm above the sediment surface. Water filtration was performed to restrict the microbial community to taxa originating from the microbial mat or the fish tissue. For anaerobic treatment, seawater was purged with N_2_ gas for 24 h before the start of the experiment. A schematic of the experimental setup can be viewed in Supplementary Figure S3.

Samples were collected on days 2, 4, 8, and 16 to monitor microbial community succession under the various experimental conditions. At each time point, two fish coupons were recovered for microscopy, while three were retrieved for microbial community profiling. Water from each container was sampled, filtered, and stored at −20 °C until analyzed.

### 2.4 Analysis

#### 2.4.1 Water chemistry

At each sampling point, pH, O_2_, and H_2_S concentrations were measured in the water column, both at the top and bottom of each mesocosm, using the Unisense UniAmp Multi Channel X-5 system (Unisense Corp., Denmark) coupled to amperometric oxygen and hydrogen sulfide microsensor electrodes, and a potentiometric pH electrode.

Dissolved inorganic carbon (DIC), dissolved organic carbon (DOC), and major ions (Ca^+2^, K^+^, Mg^+2^, Na^+^, and ${\text{PO}}_{4}^{-3}$) in the mesocosm water were analyzed at each sampling point by Chem Centre, Perth, WA. Analytical methods included total carbon combustion for DIC and DOC, microwave digestion, and Inductively Coupled Plasma-Atomic Emission Spectrometry (ICP-AES) for ion quantification, following accepted international analytical methods (APHA, USEPA, NIOSH, OSHA, ISO).

#### 2.4.2 Microbial community composition

To analyze microbial community dynamics associated with fish tissue decay over time, 16S rRNA gene amplicon sequencing was performed using both DNA and RNA templates. Upon retrieval from the mesocosms, fish tissue coupons were swabbed using sterile cotton swabs. Swabs were immediately preserved in DNA/RNA Shield™ (Zymo Research) and stored at −80 °C until extraction. Total DNA and RNA were co-extracted using the ZymoBIOMICS DNA/RNA Miniprep Kit (Zymo Research, CA, USA) following the manufacturer's protocol. Isolated DNA and RNA concentrations were quantified using the Qubit dsDNA HS Assay kit and Qubit RNA Assay kit (Life Technologies, Waltham, Massachusetts, United States). Subsequently, RNA was transcribed into complementary DNA (cDNA) using the High-Capacity cDNA Reverse Transcription Kit (Applied Biosystems™, Foster City, California, United States), according to the manufacturer's instructions.

The V3-V4 hypervariable region of the bacterial and archaeal 16S rRNA gene was amplified using the forward primer TCGTCGGCAGCGTCAGATGTGTATAAGAGACAG, and the reverse primer GTCTCGTGGGCTCGGAGATGTGTATAAGAGACAG. The primary PCR amplification was performed on a CFX Connect Real-Time PCR Detection System (Bio-Rad) using TB Green Premix Ex Taq (Tli RNase H Plus) (Takara, Japan). Each PCR reaction mix contained 1 μl of DNA or cDNA template, 0.1 μL of each primer pair (final concentration 0.25 μM), 10 μL of TB Green Premix Ex Taq, and ddH2O to a final volume of 20 μL. Thermocycling conditions were as follows: initial denaturation at 95 °C for 1 min; 42 cycles of 95 °C for 5 s, 58 °C for 30 s, and 72 °C for 60 s; followed by a final extension at 72 °C for 5 min. First-round amplicons were sequenced by the Australian Genome Research Facility (AGRF) using standard protocols for the Illumina MiSeq platform (Illumina, 2013).

#### 2.4.3 Bioinformatic workflow and statistical analysis

Raw sequence data was processed using QIIME 2 (v2020.11). Quality filtering, denoising, and chimera removal were performed using the DADA2 plugin (Callahan et al., 2016), which infers amplicon sequence variants (ASVs) based on exact sequence differences. Amplicon sequence variants (ASVs) were taxonomically classified using BLAST against the SILVA database (v138.1) (Quast et al., 2012). Downstream analyses were performed in R (v4.2.4) and RStudio (v2022.02.3). Relative abundance of bacteria at the phylum, and genus levels were calculated using the *phyloseq* package v1.40.0 (McMurdie and Holmes, 2013). Bart charts of microbial communities, including phylogenetic groups with relative abundances ≥3% in at least one sample, were created using the *ggplot2* package (Wickham, 2011).

Alpha and beta diversity metrics were calculated using the estimate_richness and evenness functions in *phyloseq* (v1.40.0). Differences in alpha diversity across time points and incubation conditions (aerobic vs. anaerobic) were assessed using analysis of variance (ANOVA), followed by Tukey's *post hoc* test for multiple comparisons (Tukey, 1949). Bray–Beta diversity was computed based on Bray–Curtis dissimilarities and visualized using non-metric multidimensional scaling (NMDS). Community structure differences were statistically evaluated using permutational multivariate analysis of variance (PERMANOVA) and analysis of similarities (ANOSIM), with significance set at *p* ≤ 0.05. The relationship between microbial community structure and water chemistry parameters was examined by fitting environmental vectors onto the NMDS ordination using the *envfit* function of the *vegan* package (Oksanen, 2016).

#### 2.4.4 Predicted functional profiles

The functional profiles from the 16s rRNA data were predicted using the Phylogenetic Investigation of Communities by Reconstruction of Unobserved States 2 (PICRUSt2) v.2.1.3-b software (Douglas et al., 2020). Kyoto Encyclopedia of Genes and Genomes (KEGG) pathway abundances (Levels 1, 2, and 3) and gene abundances were inferred from the predicted KEGG ortholog groups (KOs). In-house scripts were used to perform a linear discriminant analysis (LDA) effect size to identify KEGG ortholog groups (KOs) that served as significant biomarkers of the microbial communities developed under aerobic and anaerobic conditions. The relationship between the microbial communities' genera and the list of the key predicted genes was evaluated with the Pearson correlation coefficient in the microbiomeMaker (v1.12.2) R package (Cao et al., 2022). The bar charts and heatmap resulting from the analysis of PICRUSt2 data were illustrated using OriginPro v2024b.

#### 2.4.5 Scanning electron microscopy and X-ray energy dispersive spectroscopy (SEM-EDS)

Fish tissue coupons were analyzed using variable pressure Scanning Electron Microscopy (VP-SEM, MIRA3 XMU, Tescan) at the John de Laeter Centre, Curtin University. Images were collected at an accelerated voltage of 10 kV- 20 kV, with a working distance of 15–20 mm, and an environmental pressure of 40 Pa. This approach allowed direct visualization of samples in their hydrated, unprocessed state, eliminating the need for chemical fixation or dehydration. Elemental composition of mineral precipitates was determined using energy-dispersive X-ray spectroscopy (EDS), and spectra were processed using Aztec^®^ 5.1 software (Oxford Instruments NanoAnalysis, High Wycombe, United Kingdom).

#### 2.4.6 X-ray diffraction (XRD)

X-ray diffraction (XRD) analysis of mineral precipitates was conducted at the John de Laeter Centre, Curtin University, using a Bruker D8A X-ray diffractometer configured in Bragg–Brentano geometry. The instrument was equipped with a copper X-ray source (40 kV, 40 mA) and a LynxEye position-sensitive detector. Scans were performed over a 2θ range of 5–90°, with a step size of 0.015° and a dwell time of 0.7 s per step. Phase identification was carried out using DIFFRAC.EVA software V6.0 in conjunction with the PDF-5+ 2025 crystallographic database.

### 2.5 Statistical analysis

Statistical differences in pH, O_2_, and H_2_S concentrations across time points and incubation conditions (aerobic vs. anaerobic) were conducted utilizing PAST (V4.03). The Shapiro–Wilk test was used to assess the normality of each variable. Then, one-way analysis of variance (ANOVA) with Tukey's *post hoc* means separation test was used to test for homogeneity of variances across variables and to identify statistically significant differences among normally distributed variables. Results of statistical tests were considered significantly different at *p* ≤ 0.05.

## 3 Results

### 3.1 Characterization of mesocosm inputs

The mineral composition of the microbial mat used in the mesocosms was assessed by X-ray diffraction (XRD). The dominant mineral phases were aragonite, halite, and calcite, with minor contributions from quartz and gypsum (Supplementary Figure S2). Water chemistry analyses showed that Shark Bay seawater was alkaline (pH 8.2) and enriched in sodium (20,000 mg L^−1^), magnesium (2,270 mg L^−1^), and sulfate (500 mg L^−1^), while phosphorus concentrations were low (< 0.02mgL^−1^). A complete summary of seawater chemistry is provided in Supplementary Table S1.

### 3.2 Water chemistry evolution

**Figure 1:**
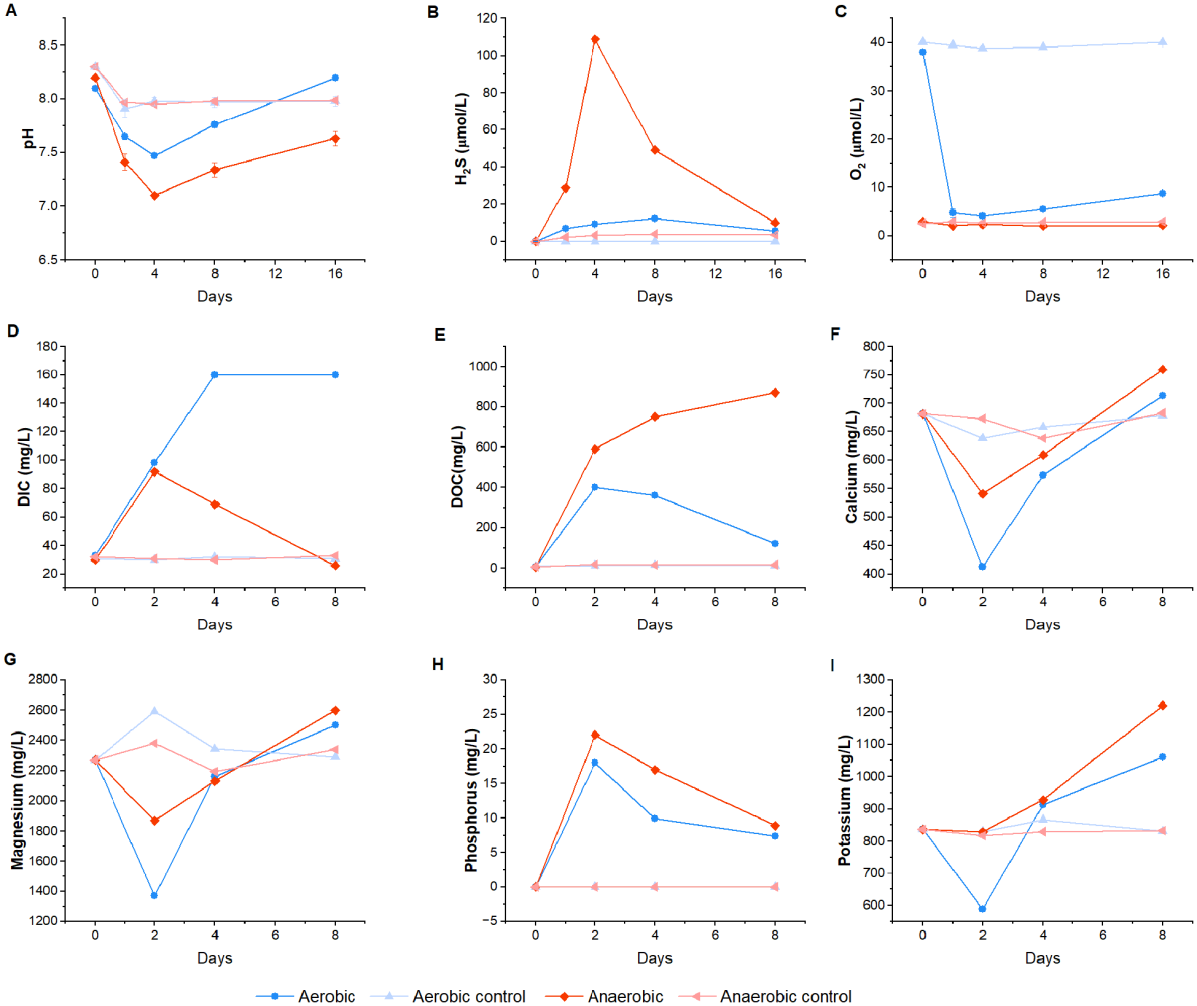
pH **(A)**, hydrogen sulfide **(B)**, dissolved oxygen **(C)**, DIC **(D)**, DOC **(E)**, calcium **(F)**, Magnesium **(G)**, phosphorus **(H)**, and potassium **(I)** concentrations measured in the water column across different experimental conditions at each sampling point. Each data point represents the mean of five independent measurements. Error bars represent standard deviations.

The temporal dynamics of pH, O_2_, and H_2_S concentrations in the water column of aerobic and anaerobic mesocosms over the 16-day incubation, measured with electrodes, are shown in Figure 1. A decline in pH was observed in both experimental and control mesocosms from day 0 to day 2, with a more pronounced drop in treatments containing fish tissue, and particularly under anaerobic conditions (Figure 1A). During this interval, pH decreased from 8.10 to 7.65 in aerobic mesocosms and from 8.20 to 7.41 in anaerobic mesocosms. The lowest pH values were recorded on day 4 (7.47 in aerobic and 7.10 in anaerobic mesocosms), after which pH gradually increased. From day 2 onwards, pH remained significantly lower in anaerobic than in aerobic mesocosms (*p* ≤ 0.05; Supplementary Table S2).

H_2_S concentrations were significantly higher in anaerobic mesocosms on days 2, 4, and 8 compared to aerobic mesocosms and controls (Figure 1B; *p* ≤ 0.05, Supplementary Table S3), peaking at 109.06 μmol/L on day 4 before returning to baseline concentration by day 16. Despite the potential for atmospheric O_2_ exchange in the aerobic mesocosms, O_2_ concentrations declined rapidly, converging with anaerobic levels by day 2 (Figure 1C). In aerobic mesocosms, O_2_ decreased from 38.04 μmol/L on day 0 to 4.69 μmol/L on day 2, reaching a minimum of 4.04 μmol/L on day 4. Anaerobic mesocosms maintained O_2_ levels between 2.02 and 3.05 μmol/L throughout the experiment. Nevertheless, O_2_ levels in aerobic mesocosms remained statistically higher than in anaerobic mesocosms (*p* ≤ 0.05, Supplementary Table 4).

pH dynamics in the mesocosm bottom mirrored those in the water column (Supplementary Figure S4), with one notable difference: from day 8 to day 16, bottom water pH remained stable at 6.5 in anaerobic mesocosms and 6.7 in aerobic mesocosms. pH values at the bottom were consistently lower than in the water column, showing a sharper decline—dropping by approximately two pH units (from −8.2 to −6.3) within 2 days, compared to a one unit decrease in the water column (from −8.3 to −7.0). The lowest bottom-water pH occurred under anaerobic conditions on day 2, in contrast to the water column, where the minimum was on day 4.

Sulfide concentrations at the bottom of the mesoosms were also markedly higher than in the overlying water (Supplementary Figure S4). Anaerobic treatments reached the highest sulfide concentration observed in the study−2,150 μmol/L on day 2—while aerobic treatments peaked at 950 μmol/L on day 8. Oxygen at the mesocosm bottom was depleted within 2 days across all treatments (Supplementary Figure S4).

Water column DIC, DOC, and major ions (Ca^+2^, K^+^, Mg^+2^, Na^+^, and ${\text{PO}}_{4}^{-3}$) diverged significantly between aerobic and anaerobic mesocosms during the incubation, reflecting fish tissue degradation dynamics (Figure 1). Both DIC and DOC increased from day 1 to day 2 in both experimental conditions (Figures 1D, E). Under aerobic conditions, DIC rose from 33 to 98 mg/L, and DOC from 7 to 400 mg/L; whereas under anaerobic conditions, DIC increased from 32 to 92 mg/L, and DOC from 6 to 590 mg/L). After day 4, the trajectories diverged. DIC under anaerobic conditions peaked at 160 mg/L (day 4) and plateaued, while DOC gradually declined to 120 mg/L by day 8. In contrast, DIC under anaerobic conditions steadily decreased to 26 mg/L by day 8, whereas DOC continued to rise, reaching 870 mg/L.

Concentrations of Ca^+2^, K^+^, and Mg^+2^ in the control mesocosms showed only minor fluctuations around their initial values (Figures 1F, G, I). In contrast, these ions declined sharply in the experimental mesocosms between day 0 and day 2. Under aerobic conditions, Ca^+2^ decreased from 681 to 423 mg/L, Mg^+2^ from 2,277 to 1,370 mg/L, and K^+^ from 837 to 588 mg/L. Under anaerobic conditions Ca^+2^ decreased from 680 to 541 mg/L, Mg^+2^ from 2,275 to 1,870 mg/L, and K^+^ from 839 to 828 mg/L; with potassium remaining relatively stable. After day 2, all ion concentrations increased substantially through day 8. ${\text{PO}}_{4}^{-3}$ concentrations showed an opposite trend (Figure 1H), rising sharply rise from 0.009 to 18 mg/L under aerobic conditions and from 0.007 to 22 mg/L under anaerobic conditions between day 0 and day 2, followed by a gradual decline; although levels remained elevated relative to the controls. Overall, ion concentrations were consistently higher in anaerobic systems than in their aerobic counterparts.

### 3.3 Microbial community dynamics under aerobic and anaerobic conditions

**Figure 2:**
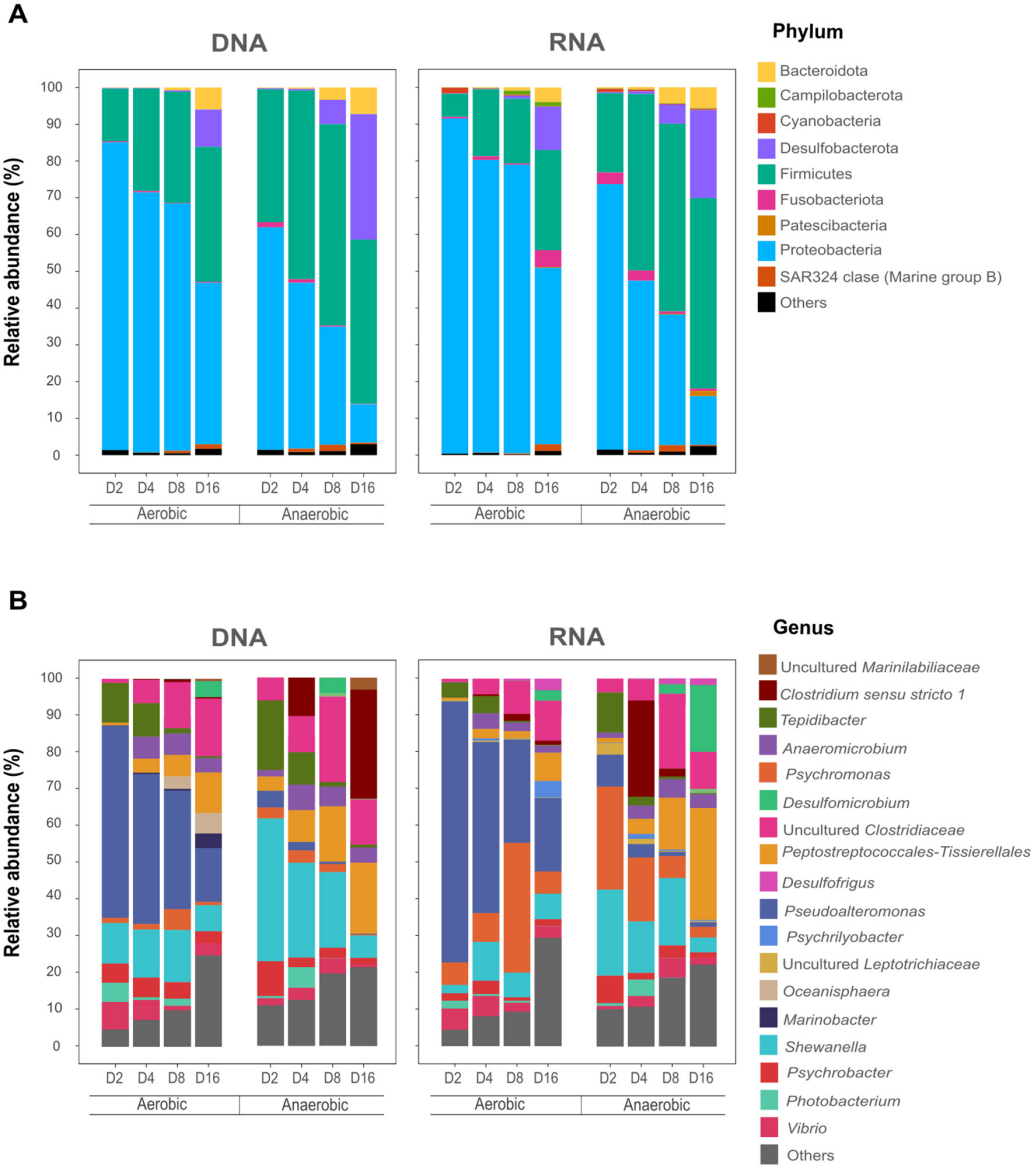
Microbial community composition at the phylum **(A)** and genus **(B)** levels during fish decay at days 2, 4, 8, and 16. DNA, DNA-based microbial profile, RNA, RNA-based microbial profile. Phylogenetic groups accounting for < 3% of all classified sequences were summarized in the group “Others”.

High-throughput 16S rRNA amplicon sequencing of microbial communities associated with fish decay under aerobic and anaerobic conditions yielded a total of 7,912,661 DNA-derived and 8,775,846 RNA-derived sequence reads. Following quality filtering, chimera removal, and singleton exclusion, 2,863,830 (DNA-based) and 3,394,689 (RNA-based) high-quality sequences were retained for downstream diversity analysis (Supplementary Table S5). Taxonomic assignment of DNA-derived reads identified 750 ASVs, encompassing 68 phyla, 125 classes, 222 orders, 303 families, and 374 genera. RNA-derived sequences revealed 720 ASVs, classified into 59 phyla, 114 classes, 206 orders, 288 families, and 362 genera. Rarefaction curves of the sequencing results confirmed sequencing depth sufficiency for all samples (Supplementary Figure S5). At the phylum level (Figure 2A), *Firmicutes* and Proteobacteria dominate the communities in both conditions; however, their relative abundances diverged markedly. Firmicutes dominated microbial communities under anaerobic conditions, whereas Proteobacteria dominated under aerobic conditions. Minor differences were observed between DNA- and RNA-derived profiles across conditions, suggesting a relatively stable relationship between total and active microbial communities.

At the genus level (Figure 2B), substantial divergence in community structure between aerobic and anaerobic treatments was observed, despite temporal shifts within each condition. RNA-derived profiles—representing metabolically active taxa—showed distinct patterns of microbial succession compared to DNA-derived profiles. Because RNA-derived data more accurately reflect metabolically active members of the community, subsequent analyses and discussion are primarily based on RNA profiles.

Under aerobic conditions, the decaying fish microbiome was initially dominated by the aerobic genus *Pseudoalteromonas*, comprising 71% of the community on day 2 and 46% on day 4. Its relative abundance declined to 28% by day 8 and 20% by day 16. A similar decreasing trend was observed for the facultative bacteria *Vibrio* and *Tepidibacter*, although their relative abundances remained comparatively low throughout the incubation. In contrast, anaerobic taxa affiliated with the Clostridiaceae, including *Clostridium* sensu stricto 1 and uncultured Clostridiaceae, were initially rare (< 1% on days 2 and 4), but increased to 1.1% and 11%, respectively, by day 16. *Psychromonas* was the second most dominant genus under aerobic conditions, maintaining 6%−8% relative abundance throughout the experiment, with a peak of 35% on day 8.

Under anaerobic conditions, no single genus maintained dominance throughout the experiment, reflecting dynamic microbial succession under sustained anoxia. The facultative anaerobic genus *Psychromonas* was initially dominant (28% on day 2 and 17% on day 4), but declined to 6% and 3% on days 8 and 16, respectively. *Pseudoalteromonas* and an uncultured Leptotrichiaceae taxon, both primarily associated with oxygenated or microaerophilic environments, followed a similar declining trend. *Shewanella*, a facultative anaerobe capable of anaerobic respiration using alternative electron acceptors (Simon, 2013; Chen and Wang, 2015), was prominent during the early and middle stages (23%, 14%, and 18% on days 2, 4, and 8, respectively), but dropped to 4% by day 16. Conversely, strictly anaerobic fermentative taxa affiliated with the Clostridiaceae family increased from ~3% on day 2 to 20% on day 8 and 10% on day 16. Similarly, members of the order Peptostreptococcales-Tissierellales, which comprise obligately anaerobic fermenters, rose from 1% on day 2 to 14% and 30% on days 8 and 16, respectively.

Initial microbiome profiles of the fish tissue (day 0) revealed that *Pseudoalteromonas* accounted for 50% of the total community, followed by *Shewanella* (29%) and *Psychrobacter* (10%) (Supplementary Table S7). In contrast, the microbial community of the surrounding mesocosms was dominated by *Coleofasciculus* (7%), Peptostreptococcales-Tissierellales (7%), and *Pseudoalteromonas* (7%). Notably, several taxa that became dominant in the fish tissue by day 16, such as uncultured Marinilabiliaceae, uncultured Clostridiaceae, *Anaeromicrobium*, and *Desulfomicrobium*, were already present at low levels in the mesocosms (1.5%, 3%, 1%, and 6%, respectively). This microbial succession occurred more rapidly under anaerobic conditions. By day 16, *Shewanella*—which initially represented 29% of the fish tissue microbiome—declined to 3% under anaerobic conditions and to 7% under aerobic conditions. Similarly, *Pseudoalteromonas*, which comprised 50% of the initial community on day 0, remained relatively abundant under aerobic conditions (20% on day 16) but was nearly absent under anaerobic conditions (~1%).

**Figure 3:**
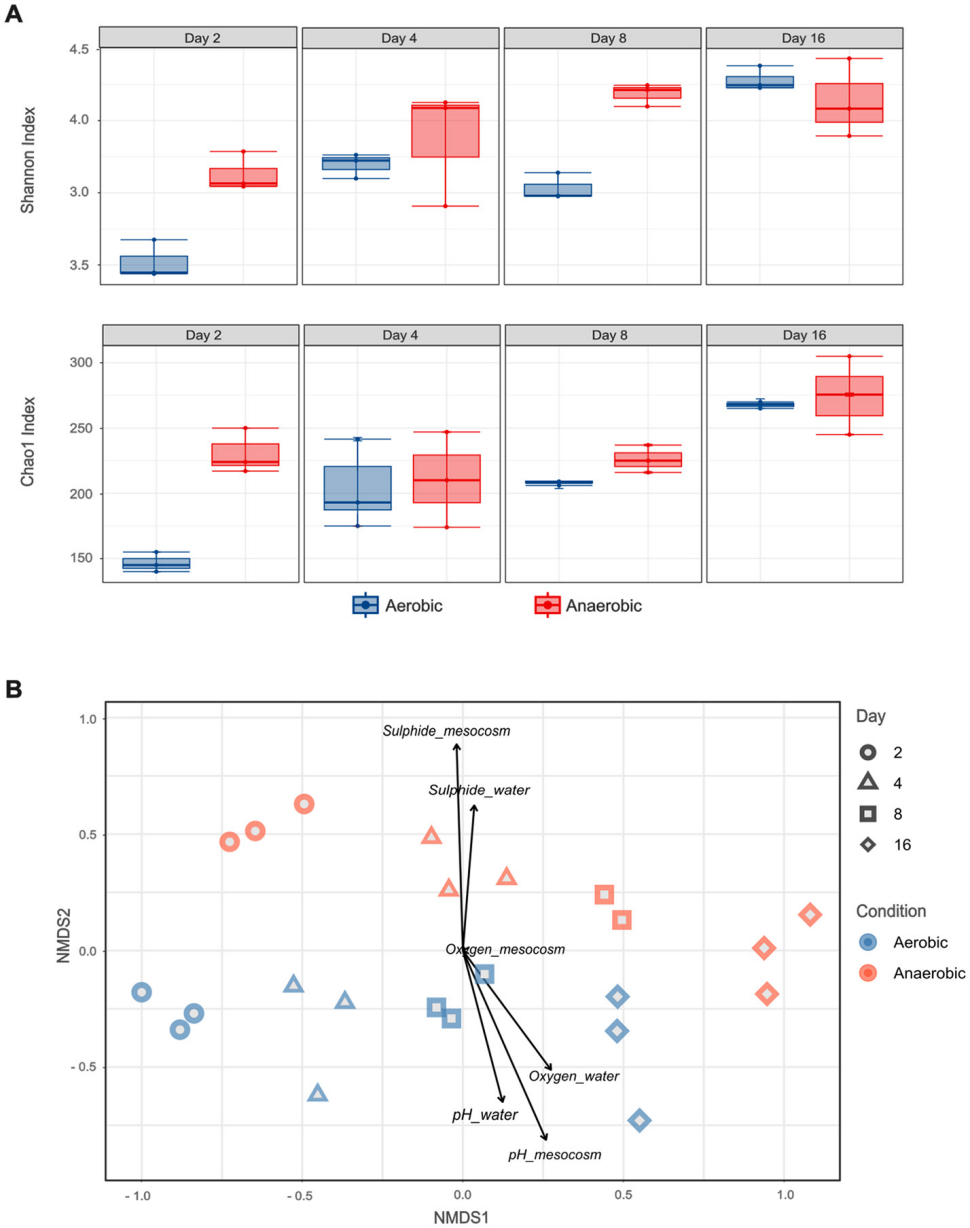
Alpha and beta diversity analyses showing differences in microbial community structure between aerobic and anaerobic conditions over time. **(A)** Shannon and Chao1 indices representing microbial diversity and richness. Boxes are extended from the 25th to the 75th percentiles, and the line in the box is plotted at the median. Whiskers represent the smallest and the largest value. **(B)** Non-metric multidimensional scaling (NMDS) based on Bray–Curtis dissimilarity, with a stress value of 0.127, illustrating temporal and condition-driven shifts in community composition.

To quantify temporal changes in microbial community composition, alpha diversity metrics—richness (Chao1 index) and diversity (Shannon index)—were calculated. Both indices consistently revealed higher diversity in communities developing under anaerobic conditions compared to those in aerobic environments, except for the Shannon index on day 16 (Figure 3A). However, statistically significant differences between aerobic and anaerobic communities (*p* ≤ 0.05, ANOVA) were observed only on days 2 and 8 for both metrics. Regardless of condition, alpha diversity increased over time, reflecting progressive diversification of the microbial community during decomposition. This trend aligns with the genus-level succession observed in the taxonomic profiles (Figure 2B), whereas initially low-diversity community dominated by a few fish-derived taxa was gradually replaced by a more diverse assemblage as mesocosm-associated microorganisms colonized the tissue and established themselves on the tissue.

Beta diversity patterns, assessed by NMDS ordination, revealed clear separation of microbial communities primarily according to incubation conditions (aerobic vs. anaerobic) (Figure 3B). This separation was statistically supported by PERMANOVA (pseudo-F = 16.49, *R*^2^ = 0.26, *p* = 0.0001) and ANOSIM (*R* = 0.48, *p* = 0.0002). Within each condition, microbial communities also clustered by time point, and pairwise comparisons confirmed that these temporal differences were significant (*p* ≤ 0.05; Supplementary Tables S8, S9).

To examine the relationship between water chemistry and microbial community structure during fish decomposition, the environmental variables— pH, O_2_, and H_2_S concentrations in both the water column and mesocosms bottom—measured via microelectrodes were fitted onto the NMDS ordination. Microbial communities in aerobic mesocosms were positively correlated with higher oxygen concentrations in the water column (*p* = 0.008) and elevated pH in both water and mesocosms bottom (*p* = 0.001 and *p* = 0.0005, respectively). In contrast, communities in anaerobic mesocosms were positively correlated with sulfide concentrations in the water and mesocosms bottom (*p* = 0.004 and *p* = 0.0005, respectively) and negatively associated with elevated pH. These findings highlight strong associations between community structure and environmental conditions, while also emphasizing that microbial activity both responds to and drives changes in water chemistry during decomposition.

### 3.4 Functional potential of microbial communities during aerobic and anaerobic decomposition

**Figure 4:**
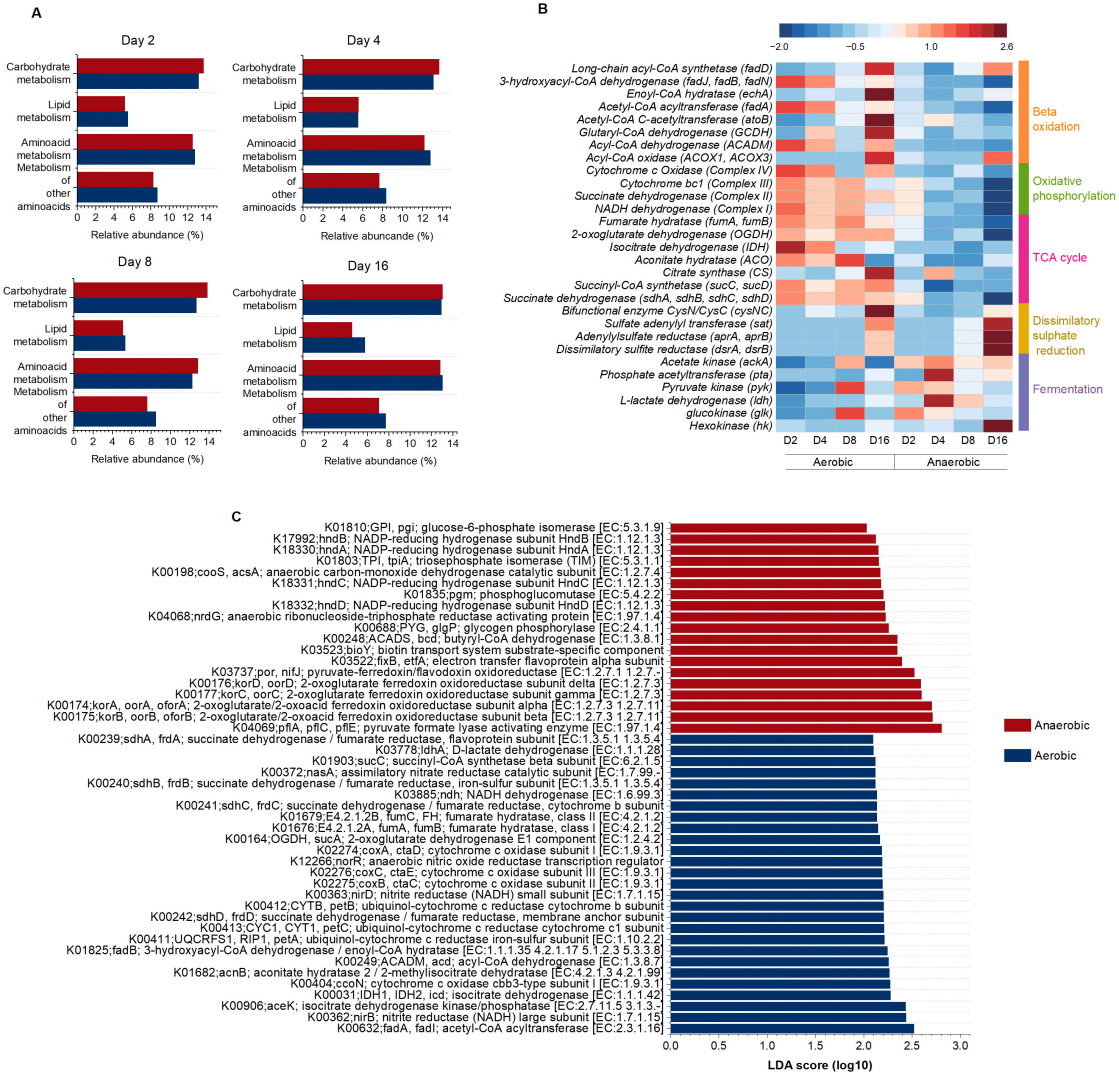
Relative abundances of predicted metabolic functions in microbial communities during fish decomposition. **(A)** KEGG Level 2 pathways within the “Metabolism” category predicted by PICRUSt2. **(B)** Heatmap showing the relative abundances of selected genes involved in fermentation, dissimilatory sulfate reduction, the tricarboxylic acid (TCA) cycle, oxidative phosphorylation, and β-oxidation of lipids in microbial communities under aerobic and anaerobic conditions. Abundances were normalized using Z-scores. Red indicates higher relative abundance, and blue indicates lower relative abundance. Color bars on the right denote the metabolic pathway associated with each gene. **(C)** Linear discriminant analysis effect size (LEfSe) analysis showing differentially abundant genes for aerobic and anaerobic microbial communities (*p* ≤ 0.05 and LDA score ≥ 2.0).

Functional profiling of microbial communities associated with fish tissue decomposition under aerobic and anaerobic conditions was performed using PICRUSt2 on 16S rRNA amplicons generated from total RNA (cDNA), emphasizing genes expressed by metabolically active microorganisms rather than dormant or inactive taxa. KEGG Level 2 pathway predictions revealed a higher relative abundance of genes associated with carbohydrate metabolism in anaerobic communities compared to their aerobic counterparts (Figure 4A). In contrast, genes involved in lipid and amino acid metabolism were more abundant under aerobic conditions. This trend persisted throughout the 16-day incubation period, indicating that, despite significant shifts in microbial community composition over time (Figure 3B), distinct and stable metabolic strategies were maintained within both aerobic and anaerobic decomposition.

Given the low resolution of KEGG Level 3 energy metabolism pathways, a targeted analysis of key energy-related enzymes predicted from the PICRUSt2-inferred metagenomes was conducted (Figure 4B). Anaerobic communities exhibited a higher abundance of genes encoding enzymes involved in fermentative metabolism (*hk, glk, ldh, pyk, pta, ackA*), and dissimilatory sulfate reduction (*dsrA, dsrB, aprA, aprB*) as energy routes, relative to aerobic communities. In contrast, aerobic communities were consistently enriched in genes associated with the tricarboxylic acid (TCA) cycle (*sucC, sucD, fumA, fumB, idh*), oxidative phosphorylation (Complexes I–IV), and β-oxidation of fatty acids (*gcdh, atoB, fadA, fadD, paaF*).

A linear discriminant analysis effect size (LEfSe) with high stringency (LDA ≥ 2 and *p-value* ≤ 0.05) was applied to the predicted metagenomes to identify the significant biomarkers of microbial communities developed under aerobic and anaerobic conditions (Figure 4C). Aerobic communities showed a significant enrichment of genes involved in fatty acid catabolism and components of the aerobic electron transport chain. These included cytochrome c oxidase subunits (K00404, K02274–76) and ubiquinol–cytochrome c reductase (K00411–13), which represent key complexes that use oxygen as the terminal electron acceptor (Kanehisa et al., 2016; Mrnjavac et al., 2024). Succinate dehydrogenase (K00239–K00242), which links the tricarboxylic acid (TCA) cycle to the respiratory chain as complex II, was also enriched, together with central TCA cycle enzymes such as isocitrate dehydrogenase (K00031) and 2-oxoglutarate dehydrogenase (K00164) that generate reducing equivalents for oxidative phosphorylation (Kanehisa et al., 2016). In addition, genes involved in β oxidation (K00632, K01825, K00249) support aerobic metabolism by supplying acetyl CoA to the TCA cycle (Clark and Cronan, 2005).

Conversely, anaerobic communities were significantly enriched in genes involved in butyrate and acetogenic fermentation, as well as anaerobic respiration. Among these, genes encoding pyruvate formate-lyase–activating enzymes (K04069; *pflA, pflC, pflE*) were enriched, consistent with the anaerobic cleavage of pyruvate into formate and acetyl-CoA via a glycyl radical mechanism (Kammel and Sawers, 2022). The enrichment of K00198 (*cooS/acsA*), encoding anaerobic carbon monoxide dehydrogenase of the Wood–Ljungdahl pathway, indicates acetogenic potential and anaerobic carbon metabolism (Ragsdale and Pierce, 2008). Butyrate fermentation was supported by the enrichment of butyryl-CoA dehydrogenase (K00248; *bcd*), a key enzyme in short-chain fatty acid production (Kanehisa et al., 2016). Finally, K03737 (*por/nifJ*), encoding pyruvate:ferredoxin/flavodoxin oxidoreductase, was enriched in anaerobic communities, reflecting a central role for reduced ferredoxin-dependent energy metabolism under oxygen-limited conditions (Ragsdale and Pierce, 2008).

These results demonstrate that aerobic and anaerobic communities differ not only taxonomically but also functionally, exhibiting distinct metabolic potentials that directly influence organic matter degradation processes.

To investigate potential taxonomic drivers of the predicted functions, Pearson correlation analyses were conducted between the relative abundances of energy-related genes and microbial taxa (Supplementary Table S10). On day 2, significant positive correlations were observed between fermentation-associated genes and genera including *Psychromonas, Tepidibacter, Clostridiisalibacter, Pseudomonas*, and *Enterococcus*. By day 4, *Clostridium* sensu stricto 7 and *Tepidibacter* were significantly associated with fermentation pathways. However, by days 8 and 16, no significant correlations were detected between fermentation-related genes and known fermentative taxa. For dissimilatory sulfate reduction, significant associations were observed with *Pseudomonas* on days 2 and 4, although these relationships were not maintained in later stages of decomposition. In contrast, aerobic communities exhibited consistent positive correlations between genes involved in aerobic respiration and β-oxidation with *Pseudoalteromonas* (days 2, 4, and 8), and with *Psychrobacter* and members of the Bradymonadaceae family by day 16.

### 3.5 Mineral precipitation

**Figure 5:**
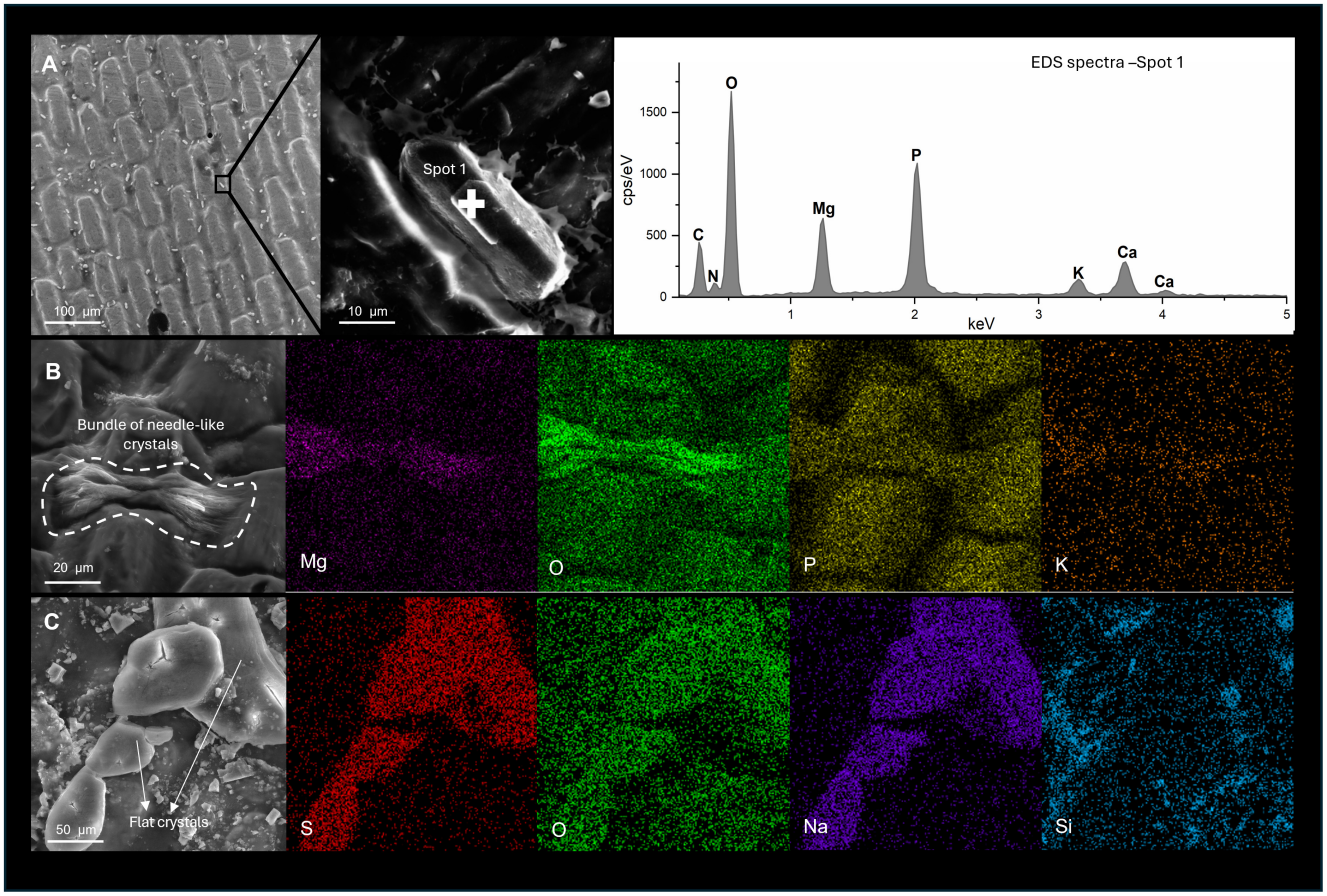
SEM images and corresponding EDS analysis of the dominant minerals observed on fish samples during the experimental period. **(A)** Overview of fish scales surface on day 0, showing abundant prismatic crystals of small size, followed by a magnified view of the marked region in A, with its respective EDS analysis showing an elemental composition of P, O, Mg in the EDS spectra. **(B)** Bundle of individual needle-like crystals with EDS elemental mapping revealing O, P, K, and Mg. **(C)** Flat, smooth crystals, with EDS elemental mapping showing O, Na, and S as the dominant elements.

SEM analyses revealed that, despite efforts to obtain fresh fish, day 0 samples were already covered by amorphous, viscous surface layers consistent with extracellular polymeric substances (EPS)—like material, and displayed small prismatic crystals on their surface (Supplementary Figure S6). EDS analysis identified these abundant prismatic crystals as enriched in Mg, O, and P, consistent with a magnesium phosphate phase (Figure 5C). Over subsequent time points under both aerobic and anaerobic conditions, the most dominant mineral phase observed were bundles of needle-like crystals, that displayed the same elemental composition as the initial prismatic crystals (Figure 5A, Supplementary Figure S7). While their overall abundance and size remained relatively stable, their morphology changed slightly over time. On day 2, the bundles consisted of thin, densely packed needles (Supplementary Figure S7); by day 4, the needles were thicker and more widely spaced; by day 8, the bundles were thinner, composed of fewer elongated crystals (Supplementary Figure S7); and by day 16, the bundles had decreased in abundance. By day 16 flat, smooth crystals were the dominant mineral phase in both conditions, EDS analysis revealed their main composition to be Na, S, and O (Figure 5C, Supplementary Figure S8). Despite these Na-S-O crystals were present on days 2, 4, and 8, they became dominant only at the end of the experiment.

Throughout the experiment, various mineral morphologies with elemental compositions indicating silicates and aluminosilicates were observed in both aerobic and anaerobic conditions (Supplementary Figure S9). These mineral morphologies and elemental compositions are consistent with those observed in the raw sediment before incubation (Supplementary Figure S10), indicating that they originated from the sediment used in the mesocosms rather than forming as a product of fish decay.

**Figure 6:**
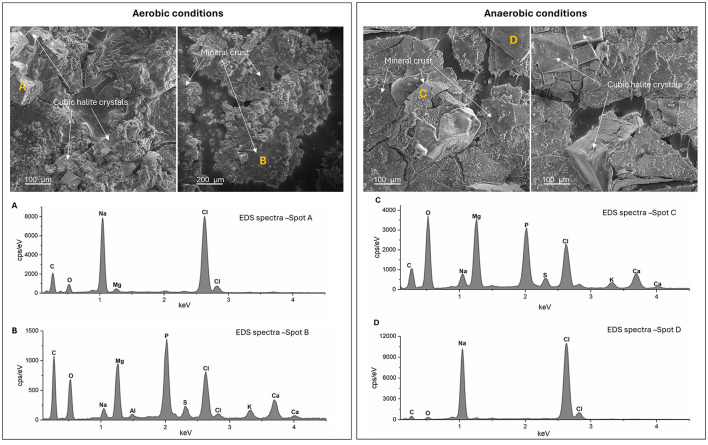
SEM images and corresponding EDS analysis of the mineral crust precipitated at the water-air interphase of experimental containers incubated under aerobic and anaerobic conditions. **(A)** EDS spectrum of a cubic crystal, indicating a composition dominated by Na and Cl, suggesting halite. **(B)** EDS spectrum of the mineral crust at point B, showing major elements Mg, P, C, and O. **(C)** EDS spectrum of the mineral crust at point C, revealing Mg, P, and O as the dominant elements. **(D)** EDS spectrum of another cubic crystal, again confirming Na and Cl as the main constituents.

In addition to the crystals on the fish samples, a continuous translucent mineral film developed at the air–water interface from day 1 onward under both aerobic and anaerobic conditions, with more extensive precipitation in aerobic systems, as evident from sampling for EDS and XRD analyses (Supplementary Figure S11). SEM-EDS analyses revealed that this crust was dominated by an amorphous magnesium phosphate phase, co-precipitating with cubic sodium chloride crystals (Figure 6). At higher magnification, smaller crystals enriched in Ca, O, and S were observed, typically associated with halite (Supplementary Figure S12) and suggesting the presence of calcium sulfate minerals, such as gypsum. The morphology of the mineral crust also differed between conditions: under anaerobic conditions, the crust consisted of overlapping, flat crystal sheets, whereas under aerobic conditions it exhibited a more aggregated, three-dimensional shape (Figure 6). XRD analysis of the crust material, after a brief rinse in cold DI water, confirmed that the dominant phase was struvite (NH_4_MgPO_4_·6H_2_O) (Supplementary Figure S13).

## 4 Discussion

### 4.1 Aerobic fish tissue decay proceeds rapidly and efficiently despite localized oxygen limitation

Microbial communities associated with fish decay under aerobic conditions followed a successional pattern dominated by Proteobacteria during the early stages of decomposition, with *Firmicutes* becoming increasingly abundant over time. This trend is consistent with previous taphonomy studies on maple leaves (Karačić et al., 2024) and crayfish (Mähler et al., 2023), forensic analyses of with vertebrate remains (Benbow et al., 2015), and human cadavers (Cobaugh et al., 2015), as well as ecological studies of leaf-litter decomposition (Zhao et al., 2017; Zhang et al., 2024). Across these systems, early decay is consistently characterized by Proteobacteria dominance, followed by a progressive increase in *Firmicutes*.

Proteobacteria dominate during the early stages likely due to their rapid growth in response to higher availability of labile organic substrates, such as sugars, amino acids, and readily degradable proteins (i.e., copiotrophic lifestyle), which are consistently available during initial organic matter degradation (Campbell et al., 2021; Hu et al., 2023). In contrast, the increasing abundance of *Firmicutes* is associated with their ecological advantages under increasingly stressful conditions (Ling et al., 2021). Many *Firmicutes* form endospores, enabling survival and metabolic activity during environmental changes, such as the pH decline, oxygen depletion, and hydrogen sulfide accumulation observed in this study (Supplementary Figure S4). Additionally, *Firmicutes* are well known to degrade more complex macromolecules, including proteins, peptides, complex lipids, and polycarbohydrates (Ling et al., 2021; Zhang et al., 2024) which accumulate after copiotrophic Proteobacteria exhaust the most labile fractions of the organic matter pool. The fact that these studies span diverse environments (e.g., freshwater, soil, and seawater) and involve a wide range of organic substrates demonstrates that early microbially mediated degradation follows a broadly predictable successional pattern.

This microbial succession likely reflects syntrophic interactions, shifts in substrate availability, and adaptation to physicochemical changes induced by preceding microbial activity. This phenomenon has been proposed as a key mechanism underlying the consistent patterns of anatomical decay observed in taphonomic experiments (Clements et al., 2025). Nevertheless, longer-term studies with detailed microbial ecological analyses are needed to fully elucidate these processes.

At the genus level, the microbial communities were dominated by *Pseudoalteromonas* and *Psychrobacter*. *Pseudoalteromonas* is commonly associated with marine environments and frequently occurs in the microbiota of fish, shrimp, and molluscs (Donovan et al., 2009; Eagan et al., 2017; Kim et al., 2024), consistent with its dominance in active fish tissue at day 0 (Supplementary Table S7). Species within this genus are linked to seafood spoilage and the development of ammonia-like off-flavors (Liang et al., 2025) and are effective organic matter degraders due to their production of diverse extracellular enzymes capable of breaking down lipids, amino acids, and proteins (Broekaert et al., 2013). Pearson correlation analyses done in this study further showed that *Psychromonas* was positively associated with genes involved in lipid β-oxidation and complete oxidation of the fish tissue.

*Psychromonas* is characteristic of highly saline aquatic environments, including polar and deep-sea waters (Lauro et al., 2013), reflecting their psychrophilic adaptations. It is frequently implicated in the spoilage of refrigerated seafood (Chen et al., 2019; Liu et al., 2023). Shelf-life studies on American lobster indicate that *Psychromonas* isolates exhibit pronounced lipolytic activity (Tirloni et al., 2016), and genomic analyses of *Psychromonas ingrahamii* reveal a facultatively anaerobic metabolism with a preference for amino acids over sugars as carbon and energy sources (Riley et al., 2008). These traits align with the functional prediction observed during aerobic decay, where pathways related to lipid and amino acid metabolism were prominent, suggesting efficient oxidation of fish tissue-derived substrates.

The Chao1 and Shannon indices increased over time, indicating that microbial diversity was higher by day 16 (Figure 3A). This contrasts with most previous decay studies (Wei et al., 2018; Miguel et al., 2021; Mähler et al., 2023). The difference is likely due to the presence of the highly diverse external inoculum provided by the microbial mat in our mesocosms, which was absent in those earlier studies. Consistent with this, 16S rRNA data show that during the initial stages of decomposition, the native fish-associated microbiota predominated. However, as decomposition progressed, low-abundance mesocosm-derived microorganisms progressively colonized the tissue and eventually displaced the initial fish-associated community (Supplementary Table S7). Similarly, in decay experiments on Australian sea urchin embryos, 16S rRNA data showed that embryos incubated with only seawater as the microbial inoculum exhibited substantially lower microbial diversity after 5 days than those incubated with a mud inoculum (Raff et al., 2008). These results underscore the influence of external inoculum on community assembly in taphonomy experiments and demonstrate that alpha-diversity trajectories during decay are not universal but depend on the interplay among microbial ecological context, physicochemical gradients, and system-specific factors.

Despite continuous oxygen exchange with the atmosphere, oxygen concentrations near the fish tissue in mesocosms and in the overlying water were significantly low throughout the experiment. However, statistical analysis of the functional predictions (LEfSe) of active microbial communities indicated that aerobic respiration was the dominant energy production pathway, and that it was supported by an enrichment of β-oxidation genes (linked to *Pseudoalteromonas*). These findings are consistent with previous taphonomy studies reporting near-zero oxygen around decaying organisms, including frogs, fish, and shrimps, within 24 h (Briggs and Kear, 1994; Sagemann et al., 1999; Iniesto et al., 2015, 2017), attributed to intense heterotrophic aerobic respiration. Although aerobic metabolism appears contradictory under near-zero oxygen, the mesocosms function as seafloor sandy sediments, oxygen in the overlying water column diffuses into the sediment and is rapidly consumed by microbes degrading organic matter (Ahmerkamp et al., 2017), creating bulk anaerobic conditions. The thin (~1.5 cm) sand layer covering the fish likely allowed rapid oxygen depletion near the surface (Jørgensen et al., 2022). Additionally, the sand matrix, owing to its grain size and permeability, can generate microscale oxygen-enriched areas (Ziebis et al., 1996; Huettel et al., 2014), producing localized aerobic microenvironments that allow organic matter oxidation, often undetectable by conventional oxygen probes due to their limited spatial resolution and sensitivity (Gäb et al., 2020).

Water chemistry and the mineral precipitates observed support the interpretation of active aerobic respiration despite low oxygen concentrations. Conceptually, aerobic microbes oxidize organic matter, producing carbon dioxide and water, a process that increases the DIC pool in the system (Kirchman, 2012). Consistent with this, DIC concentrations were higher under aerobic than anaerobic conditions (Figure 1D), indicating more complete oxidation of organic matter to CO_2_ (Hoffmann et al., 2021). Beyond CO_2_ production, organic matter degradation releases other key elements into the surrounding environment. In vertebrates, carbon constitutes roughly 20% of total body mass, while calcium, nitrogen, phosphorus, sulfur, sodium, and iron are present in smaller amounts (DeBruyn et al., 2025). During active decomposition, these elements are rapidly mobilized. Previous studies reported an 80% increase in total nitrogen and phosphorus after 4 days of rabbit carcass decay (Quaggiotto et al., 2019) and 3.7–22-fold increases in Na, S, P, and K during human decomposition (Taylor et al., 2023).

In our experiment, major ion concentrations were consistently lower under aerobic conditions (Figures 1E–I). While this might initially suggest slower decay relative to anaerobic conditions, it is important to consider that mineral dissolution and precipitation are governed by chemical equilibrium, with precipitation occurring when the water becomes oversaturated with respect to a mineral phase (Hoffmann et al., 2021). The more extensive formation of mineral precipitates (mainly struvite) at the air–water interface under aerobic conditions (Supplementary Figure S11) indicates that oversaturation of Mg^+2^ and ${\text{PO}}_{4}^{-3}$ occurred more rapidly, reducing the concentration of free ions in the water, rather than reduced element mobilization. Together with the elevated relative abundance of genes associated with amino acid and lipid metabolism, these observations indicate that decomposition rates were higher under aerobic conditions than in anaerobic conditions.

### 4.2 Anaerobic fish tissue decay proceeds more slowly through fermentative pathways and incomplete mineralization

Under anaerobic conditions, microbial community succession during fish decay followed a consistent trajectory characterized by an early dominance of Proteobacteria, followed by a progressive increase in *Firmicutes*, together with contributions from Bacteroidota and Desulfobacterota at later stages. This succession mirrors patterns observed in previous decay studies (Lobb et al., 2020; Mähler et al., 2023; Karačić et al., 2024) and likely reflects shifts in substrate availability and redox conditions during anaerobic decomposition.

The early dominance of Proteobacteria in organic matter decay reflects their copiotrophic lifestyle (as described in Section 4.1); however, establishing a nutrient-rich environment depends on their ability to extracellularly hydrolyze proteins and peptides into smaller molecules (Liu and Liu, 2020). In the absence of oxygen, these Proteobacteria likely contributed to the organic degradation primarily via enzymatic lysis of macromolecules rather than direct oxidation of labile substrates (Tang and Manefield, 2025). The subsequent rise of *Firmicutes* corresponds to their ability to thrive in anaerobic, nutrient-enriched environments generated by prior Proteobacterial activity. Under anoxic conditions, many *Firmicutes* can hydrolyze and ferment a broad range of organic substrates (Tonanzi et al., 2021) and degrade complex carbohydrates (Zhang et al., 2024). Their increased abundance, together with the higher relative abundance of fermentation-associated genes in anaerobic compared with aerobic communities, indicates a metabolic shift from primary macromolecule hydrolysis toward fermentation-based energy conservation during anoxic organic matter degradation.

Anaerobic decomposition also seems to promote syntrophic interactions between *Firmicutes* and Bacteroidota, where *Firmicutes* perform primary hydrolysis of complex macromolecules, and Bacteroidota converts labile substrates into acetate, butyrate, and propionate (Hu et al., 2024; Baldasso et al., 2025). The later emergence of Desulfobacterota, which includes most known sulfate-reducing bacteria (SRB) (Waite et al., 2020), reflects their reliance on the availability of low-molecular-weight fermentation products such as acetate, lactate, propionate, and hydrogen as electron donors, generated primarily by Proteobacteria and *Firmicutes* during organic matter breakdown (Jørgensen et al., 2019).

At the genus level, the community shifted from early dominance by *Psychromonas* to later prevalence of members of the bacterial family Clostridiaceae thereafter. As discussed in Section 4.1, *Psychromonas* is commonly associated with fish spoilage through lipolytic activity and amino acid metabolism. Its early dominance and correlation with fermentation (Supplementary Table S10) likely reflects its role as a primary degrader, generating low-molecular-weight substrates that support the growth of fermentative taxa such as *Tepidibacter*. Although *Tepidibacter* has not previously been reported in fish decay, most described species are fermenters capable of degrading glucose, amino acids (e.g., alanine and proline), and complex organics such as peptone and yeast extract (Slobodkin et al., 2003; Urios et al., 2004; Tan et al., 2012; Dai et al., 2023), suggesting they can utilize hydrolysis products released by *Psychromonas*.

Members of the family Clostridiaceae were also positively associated with fermentation (Supplementary Table S10). This ubiquitous family comprises different putrefactive bacteria (Miguel et al., 2021), capable of degrading carbohydrates, proteins, and long-chain fatty acids into smaller metabolites that serve as substrates for other microbes (Paczkowski and Schütz, 2011; Hu et al., 2024). *Clostridium* species are frequently implicated in early fish decay (Lobb et al., 2020) and are relevant to taphonomy, as their incomplete lipid hydrolysis is considered a key mechanism underlying exceptional fossil preservation (Mähler et al., 2022). The absence of β-oxidation genes in the anaerobic microbial community further demonstrates limited lipid degradation under anoxic conditions.

In addition to fermentative taxa, *Shewanella* constituted an important component of anaerobic decay communities. Several species of this genus have been isolated from spoiled fish (Zhang et al., 2022) and are known for producing the characteristic “fishy” off odor of decomposing seafood. During anaerobic respiration, *Shewanella* can use trimethylamine-N-oxide (TMAO), an abundant osmolyte in fish muscle, as a terminal electron acceptor, reducing it into trimethylamine (TMA), generating energy in the process and contributing to the odor (Lou et al., 2021).

Water chemistry measurements revealed a rapid decline in pH within the first 2 days of decay in both aerobic and anaerobic treatments, at both the water column and mesocosm bottom (Figure 1A, Supplementary Figure S4A). However, anaerobic conditions consistently produced more acidic environments than aerobic ones, consistent with previous decay and taphonomy experiments (Briggs and Kear, 1993a,b, 1994; Miguel et al., 2021). (Briggs and Kear 1993a) reported a drop in pH after 2 days, reaching 6.7 in open vessels and 5.32 under anaerobic conditions, attributing the difference to retention of CO_2_ and fatty acids under anoxia, implying the predominance of fermentative metabolism in oxygen-depleted systems. Our PICRUSt2 and LEfSe analyses support this interpretation; anaerobic communities were enriched in carbohydrate metabolism pathways, with KEGG Orthologs essential for fermentation identified as biomarkers (Figure 4C). Specifically, genes for butyric acid and acetic acid production. Fermentative metabolism generates volatile fatty acids such as acetate and butyrate, which have lower pKa values than carbonic acid and therefore contribute more strongly to proton release and pH decline (Pines et al., 2016). In contrast, carbon dioxide produced under aerobic conditions is readily lost to the atmosphere and exerts only weak acidifying effects on the aqueous environment (Solis-Herrera and Carmen, 2024).

Volatile fatty acids (VFAs) generated during anaerobic decay accumulated in pore waters because the microbial community lacked the terminal electron acceptors and metabolic pathways necessary for their further oxidation. In contrast, aerobic metabolism fully mineralized organic carbon to CO_2_, resulting in less acidic conditions. This difference is reflected in consistently higher DOC and lower DIC concentrations under anaerobic conditions compared to aerobic decay. Fermentation also yields substantially less energy (~244 kJ·mol^−1^) than aerobic respiration (~3,000 kJ·mol^−1^) (Schoenen and Schoenen, 2013), suggesting slower microbial growth and activity under anoxia. Supporting this mineral precipitation at the air–water interface was reduced in anaerobic treatments (Supplementary Figure S11), and relative abundances of genes associated with amino acid and lipid metabolism were lower. The slower accumulation of Mg^+2^ and ${\text{PO}}_{4}^{-3}$ required for struvite formation further indicates reduced decay rates in the absence of oxygen.

### 4.3 Mineral formation and implications for the fossilization process

Microsensor analyses revealed that, regardless of bulk aerobic or anaerobic conditions, the microenvironment surrounding decaying fish tissue remained chemically distinct from the overlying mesocosm water for up to 16 days (Figure 1, Supplementary Figure S4). On day 2, bottom-water pH was around 1 unit lower than the water column (6.5 vs. 7.6) under aerobic conditions, and 1.4 units lower under anaerobic conditions (6.0 vs. 7.4). By day 8, H_2_S concentrations near the carcasses had increased by an order of magnitude relative to the overlying water in the anaerobic treatments (1,500 vs. 150 μmol·mL^−1^), and nearly 80-fold under aerobic conditions (1,000 vs. 12 μmol·mL^−1^). Such steep chemical gradients have been observed in previous taphonomy experiments and are closely linked to localized mineral precipitation (Sagemann et al., 1999; Vietti et al., 2015; Naimark et al., 2016).

In this study, struvite (NH_4_MgPO_4_·6H_2_O) was the predominant mineral phase formed on decaying fish tissue under both aerobic and anaerobic conditions, indicating that, despite differences in bulk water chemistry, microbial community composition, and metabolic potential, the immediate microenvironment around the tissue became oversaturated with the same suite of ions, driven primarily by the tissue's own composition. Moreover, our results demonstrate that during organic matter decay, microorganisms facilitate mineral formation at the micrometric scale by creating localized microenvironments distinct from the bulk. Although equilibrium-chemistry models predict struvite precipitation at 7.0–11 (Taylor et al., 1963; Loewenthal et al., 1994), we observed crystals formed in the pore waters of pH 6.0 to 6.6. This implies the formation of pH-elevated microzones driven by microbial activity, consistent with bacteria-induced mineral precipitation (BIMP) (Hoffmann et al., 2021). In this process, metabolic by-products and microbial surface interactions create fine-scale chemical heterogeneity that controls mineral nucleation, independent of bulk water chemistry. Comparable microscale mineralization patterns, in which minerals are tightly associated with organic matter and microbial structures, have been widely documented across diverse depositional environments and fossil deposits (Stevens et al., 2015; Cabestrero et al., 2018; Dias and Carvalho, 2022).

Exceptionally preserved soft tissues in the fossil record are commonly associated with calcium phosphate mineralization, which can capture fine anatomical detail (Wilson et al., 2016). Closed-system decay experiments using shrimp in artificial seawater have reported rapid calcium phosphate (apatite) precipitation within 2 weeks (Briggs et al., 1993; Briggs and Kear, 1993c). In contrast, struvite was the dominant phase in our system (Figure 6, Supplementary Figures S5, S10). This divergence is most plausibly explained by the higher magnesium availability and the persistently high Mg/Ca ratio (>5) characteristic of our system (Figures 1F, G). The magnesium concentration of Shark Bay water (2,270 mg·L^−1^; Supplementary Table S1) was nearly twice that of the artificial seawater used in the earlier studies [(Briggs and Kear 1993c) 1,277 mg·L^−1^]. High magnesium concentrations are well known to inhibit calcium phosphate nucleation while promoting magnesium phosphate formation (Salimi et al., 1985; Jiang et al., 2021). Mechanistically, magnesium interferes with apatite mineralization via two principal pathways: (i) substitution of Ca^+2^ in the apatite lattice, which distorts crystal structure and prevents the development of a well-crystalline phase, thereby blocking continued deposition; and (ii) stabilization of more soluble or metastable phosphate phases (Cao et al., 2007). Together, these effects provide a clear geochemical explanation for the prevalence of struvite rather than apatite in our decay system.

Although magnesium phosphate minerals, such as struvite, are not among the minerals typically associated with fossil preservation (Farrell, 2014; McCoy, 2014; Schiffbauer et al., 2014), they are known to form transiently under alkaline conditions enriched in ammonia, magnesium, and phosphate; in environments such as wastewater systems, old cemeteries, animal waste deposits, and decomposing cartilage (Rogers et al., 2014; Liang et al., 2024). This implies that struvite may form commonly—but only briefly during the early stages of decomposition, especially in magnesium-rich tissues.

Because struvite stability is highly sensitive to pH, temperature, and ion oversaturation (Roncal-Herrero and Oelkers, 2011)—variables that fluctuate during diagenesis (Barnes et al., 1984)—we propose that, under favorable diagenetic conditions, struvite dissolution may liberate phosphate that subsequently participates in the precipitation of more stable minerals such as apatite, ultimately contributing to long-term soft tissue preservation. This interpretation is consistent with previous suggestions that the absence of struvite in ancient deposits is due to its transformation into more stable phases (e.g., calcite or gypsum) rather than its original absence (Feng et al., 2020).

In addition to magnesium phosphates, tabular Na-, S-, and O-enriched crystals were observed throughout the experiment and became dominant by day 16 (Figure 5C, Supplementary Figure S8). Based on their elemental composition and the chemistry of the system, these crystals are most consistent with sodium sulfate evaporites. Evaporites are sedimentary minerals that form when saline waters—such as those in tidal flats or hypersaline lakes—undergo evaporation, producing ion-saturated brines from which minerals precipitate (Tiab and Donaldson, 2016). Their composition typically reflects that of the parent solution, containing variable proportions of Na, Ca, Mg, K, Cl, SO_4_, and CO_3_, as well as trace elements (e.g., B, Ba, Sr, Br, Li, I) (Warren, 2010). Although definitive mineral identification by XRD or thermogravimetric analysis (TGA) was not possible due to the micrometric scale of these crystals, our water chemistry data indicate that decay-driven ion release generated oversaturation states consistent with evaporitic conditions. Comparable sodium sulfate minerals, such as mirabilite (Na_2_SO_4_·10H_2_O), and its dehydrated form, thenardite (Na_2_SO_4_), have been documented in shallow saline lakes colonized by microbial mats in Central Spain (Cabestrero et al., 2018), forming during warm periods when microbial mat decay and high evaporation drive oversaturation of Mg^2^^+^-SO_4_^2^^−^-Cl^−^ brines. Although sulfate was not directly quantified in our water chemistry analyses, the predicted microbial functional profiles and mineralogical evidence suggest increasing sulfate concentrations toward the end of the experiment. The dominance of these Na-, S-, and O-enriched crystals by day 16 corresponds to a higher predicted abundance of dissimilatory sulfate-reduction genes, indicating that sulfate was available in sufficient concentrations during late decay. Coupled with the initially high chloride and magnesium levels in Shark Bay water, we infer that the observed mineral phase corresponds to mirabilite or its dehydrated counterpart, thenardite.

Fossil occurrences associated with sulfate evaporitic minerals are rare but notable (Plet et al., 2017; Elson et al., 2025). For instance, adult frogs (*Rana pueyoi*) from the Libros lacustrine system (early Miocene–Pliocene) show abundant gypsum (CaSO_4_·2H_2_O) discoids associated with preserved skin (McNamara et al., 2009). Likewise, Upper Cretaceous bone remains from Lo Hueco (Cuenca, Spain) exhibit gypsum infilling within spongy cavities and precipitation on external bone surfaces (Cambra-Moo et al., 2012). Experimental evidence for gypsum precipitation during decay is limited to a single study by (Iniesto et al. 2018), who reported gypsum forming on both the surface and internal tissues of fern pinnae after 60 days of incubation within microbial mats.

In contrast, our study provides the first report of abundant sodium sulfate mineral precipitation in a taphonomy context, revealing that other evaporite phases may also contribute to early mineralization. Mirabilite and thenardite (Na_2_SO_4_), have recently been demonstrated to entomb diverse microorganisms and organic compounds, such as β-carotene, in the Na_2_SO_4_-saturated springs of the Great Salt Lake (UT, USA) during winter (Gill et al., 2023). Therefore, analogous to our proposal that struvite may act as a precursor to more stable minerals, we propose that mirabilite may serve as a precursor to gypsum during fossilization, with environmental changes driving these transformations. Such transformations among sulfate minerals are consistent with mineralogical and sedimentological evidence from natural evaporite basins and diagenetic studies (Ortí et al., 2002; Herrero et al., 2015).

## 5 Conclusions

The chemical and microbiological changes occurring during fish-tissue decomposition under aerobic and anaerobic conditions, and their implications for mineral formation, were investigated. The main findings are:

° Aerobic and anaerobic conditions produced differentiated microbial communities that remained distinct throughout the 16-day incubation. Anaerobic mesocosms supported a more diverse community, positively associated with lower pH and elevated hydrogen sulfide concentrations, whereas aerobic treatments supported communities associated with higher pH and greater oxygen availability. Although the two treatments followed different successional trajectories, their predicted metabolic functions at the KEGG pathway level were broadly similar from day 2 onward.
° Higher DIC and lower DOC characterized aerobic conditions, consistent with more complete oxidation of organic carbon to CO_2_ than that in anaerobic conditions. Correspondingly, the aerobic microbial community exhibited higher relative abundances of genes associated with aerobic respiration and complete mineralization of organic matter. *Pseudoalteromonas* was highly abundant in these treatments and was positively correlated with β-oxidation pathways of fatty acids, which sustain aerobic respiration during fish-tissue degradation.
° Anaerobic treatments exhibited higher DOC, lower DIC, and lower pH relative to aerobic conditions. These patterns are consistent with higher relative abundances of carbohydrate metabolism and fermentation pathways in anaerobic communities. Members of the family Clostridiaceae were significantly associated with these metabolic functions. Reduced mineral precipitation at the water–air interface under anaerobic conditions indicates less extensive tissue degradation, consistent with the lower energy yield and incomplete breakdown typical of fermentation.
° Regardless of redox conditions, magnesium phosphates and sodium sulfates were the primary minerals formed. We propose that these minerals represent early-stage products of fish fossilization that may undergo subsequent diagenetic transformation into more stable mineral phases commonly observed in fossil deposits.

## Data Availability

The datasets presented in this study can be found in online repositories. The names of the repository/repositories and accession number(s) can be found below: https://www.ncbi.nlm.nih.gov/, PRJNA1377284.
