# Supplementary material for: Microbial dynamics and metabolic activity during fish decay under aerobic and anaerobic conditions: insights into microbial fossilization
Source: Front Microbiol. 2026 Apr 16;17:1783163. doi: 10.3389/fmicb.2026.1783163 (PMC13128626; doi:10.3389/fmicb.2026.1783163)
Supplement: Supplementary file 2 [file Table_1.docx]

Supplementary Material

# Supplementary Figures


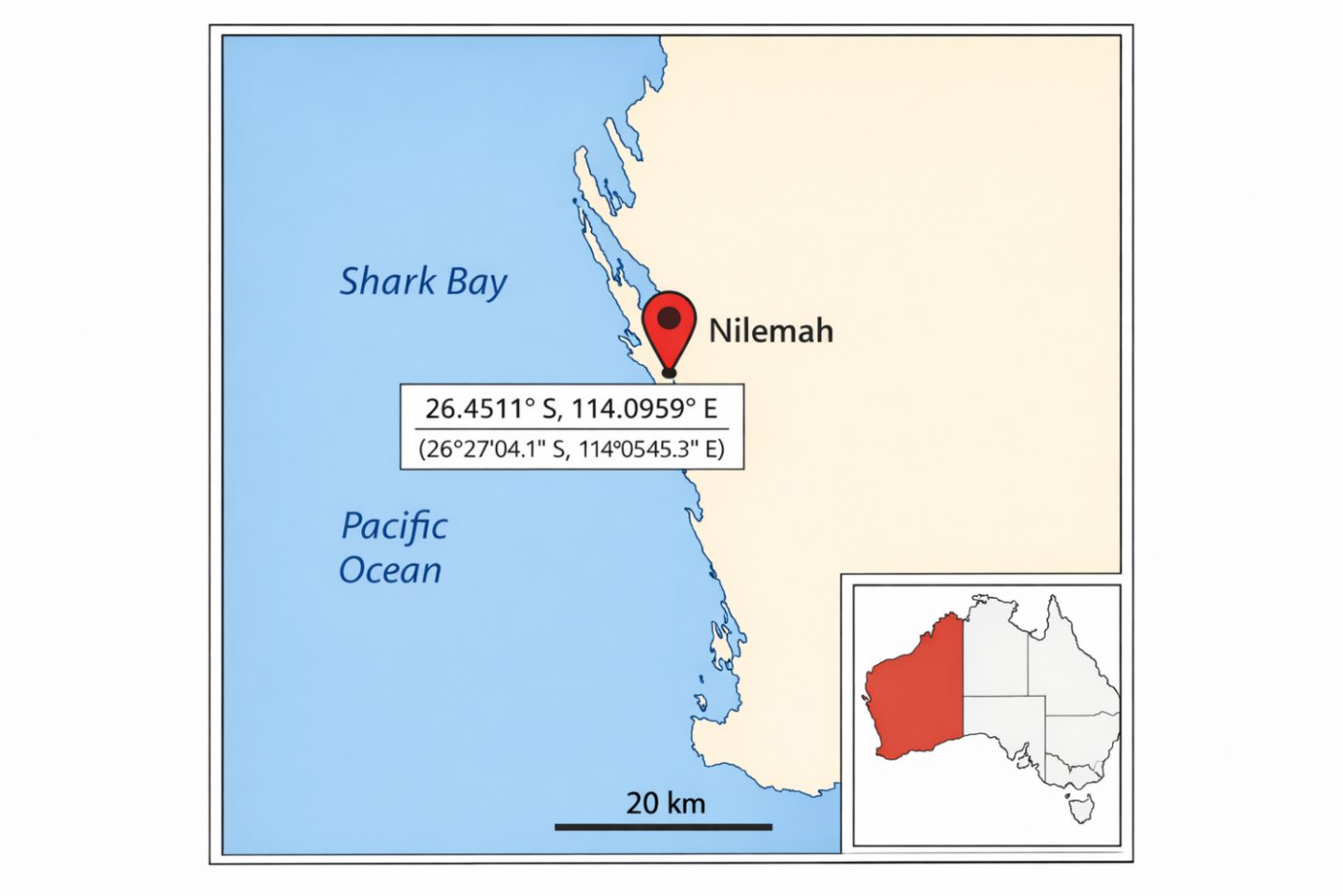


**Figure S1.** Map of the Nilemah region in Shark Bay, Western Australia, indicating the microbial mat collection site (26°27′04.1″ S, 114°05′45.3″ E).





## Figure S2. XRD diffractogram of the freeze-dried microbial mat. Gy, gypsum (CaSO4.2H2O); Qz, quartz (SiO2); Ar, aragonite (CaCO3); Ca, calcite ((Ca,Mg)CO3); Ha, halite (NaCl).


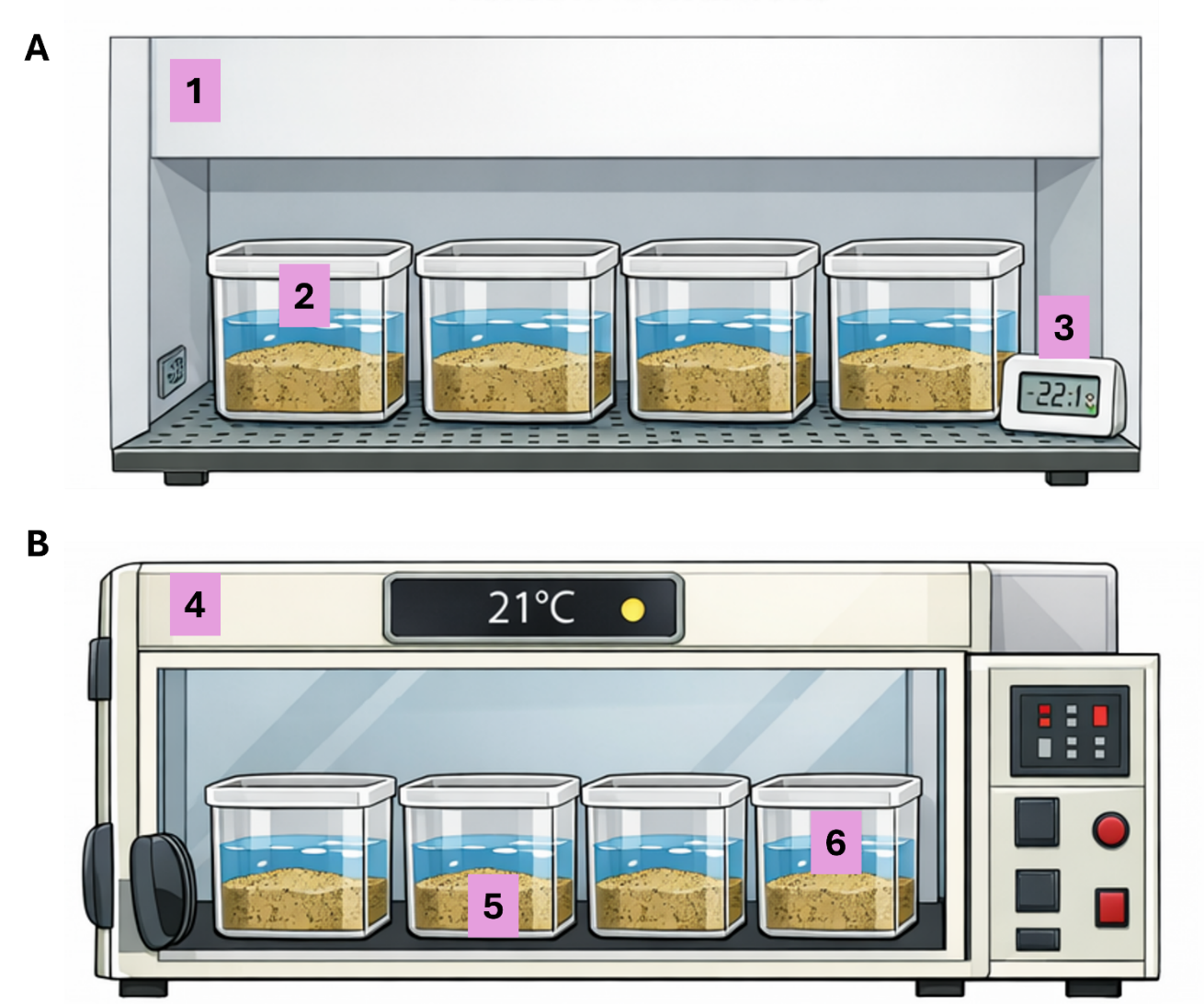


**Figure S3.** Experimental setup for studying fish tissue decay under aerobic (A) and anaerobic (B) conditions. The setup includes: (1) a fume hood for aerobic incubations; (2) squared plastic containers used to contain the fish tissue, sediment, and seawater; (3) a portable temperature monitor used to track ambient temperature under aerobic conditions; (4) an anaerobic chamber maintained at approximately 21 °C for anaerobic incubations; (5) fish tissue pieces placed at the bottom of each mesocosm and completely buried; and (6) sterile seawater collected from Shark Bay used as the overlying water column.





## Figure S4. pH (A), hydrogen sulphide (B), and dissolved oxygen (C) concentrations measured at the bottom of the mesocosm at each sampling point during the experiment. Each data point represents the mean of five independent measurements.


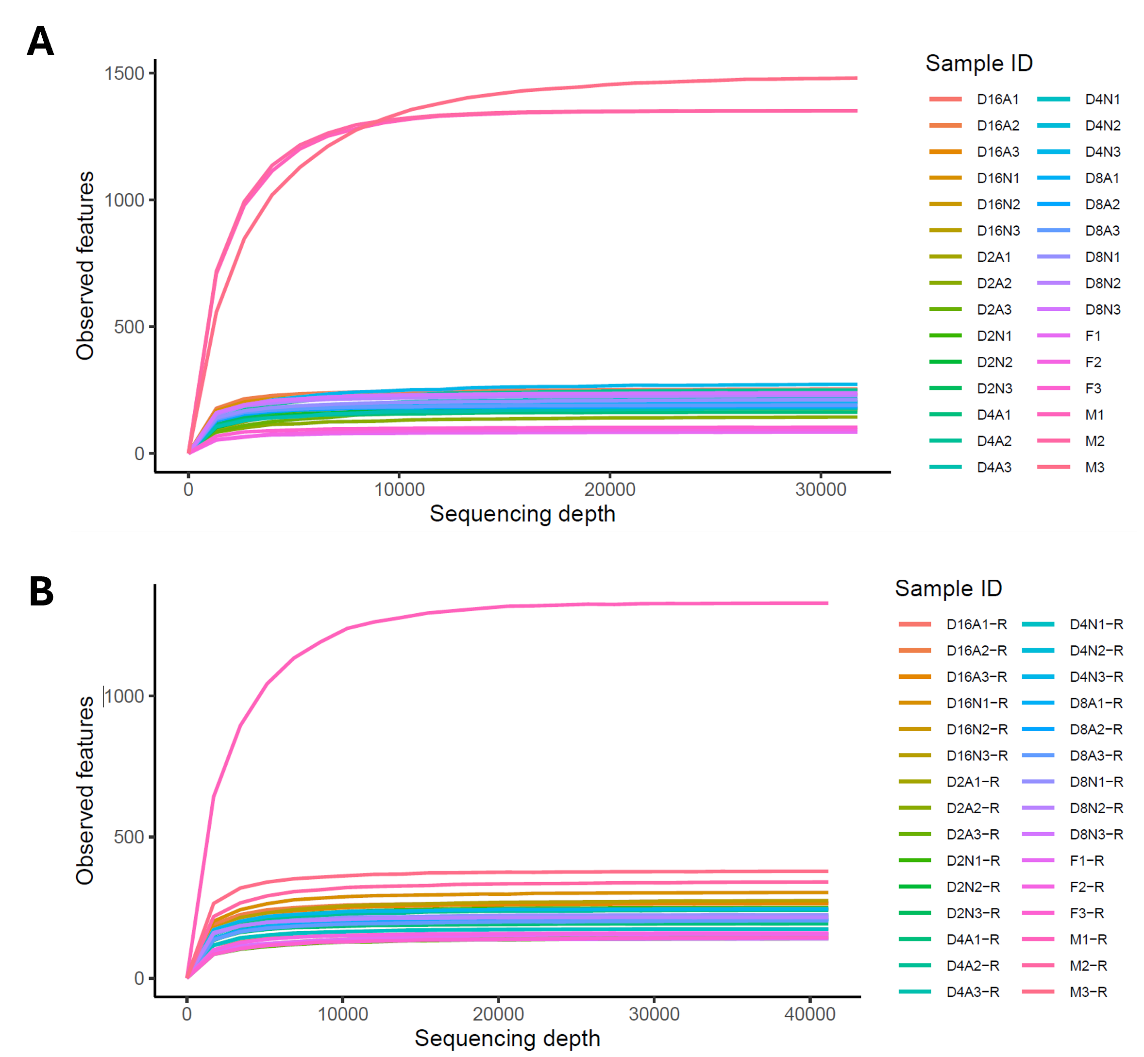


## Figure S5. Individual alpha rarefaction curves (Observed features) for all samples. DNA-based sequencing (A), RNA-based sequencing (B).


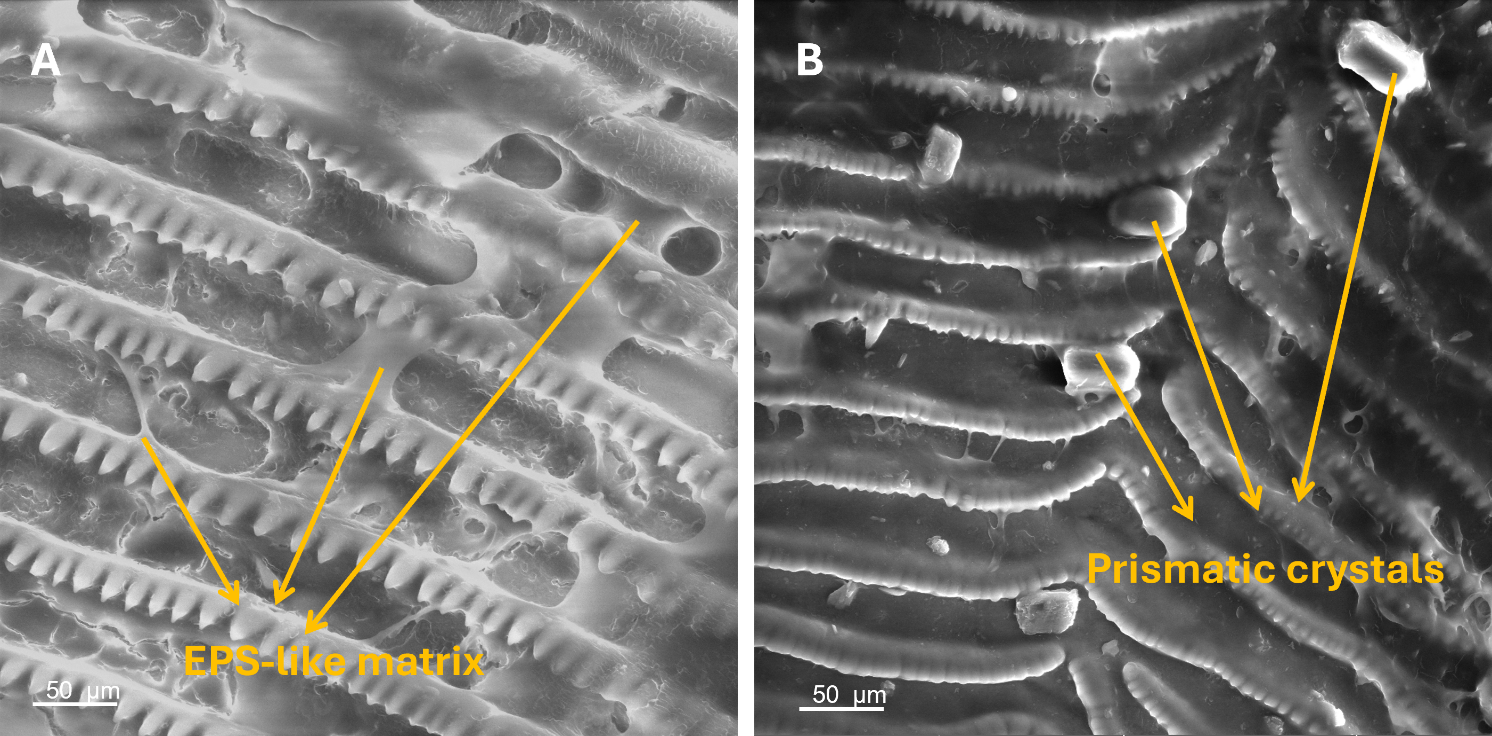


**Figure S6**. SEM image of the fish scales on day 0, covered in EPS-like structures (A) and prismatic crystals (B).


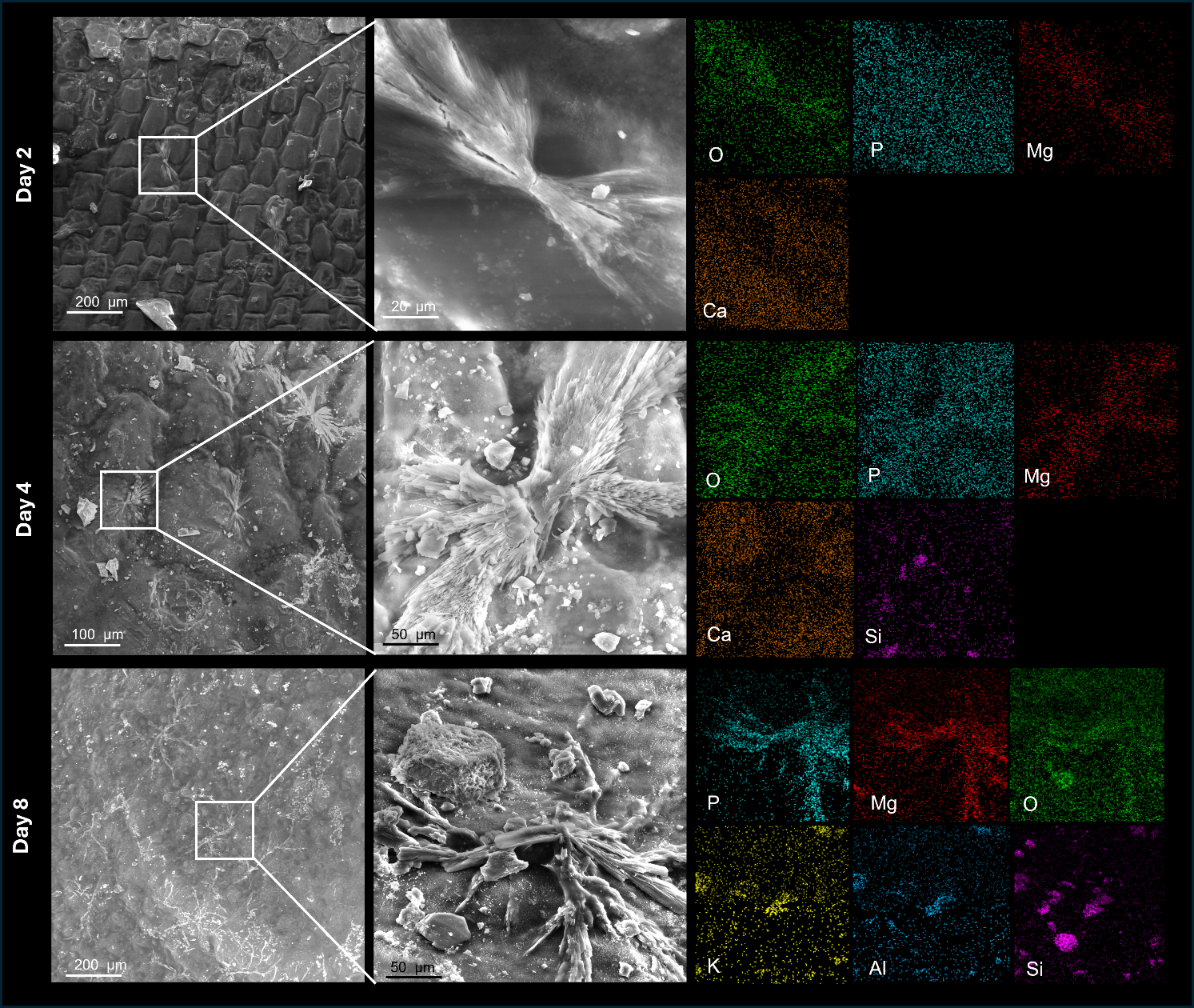


**Figure S7**. SEM micrographs and corresponding EDS elemental maps of mineral bundles or needle-like individual crystals observed on fish samples at days 2, 4, and 8. The first column shows low-magnification images illustrating the overall sample coverage of the crystals. The second column presents higher-magnification views highlighting the morphology of the bundles. The third column displays EDS maps, revealing oxygen, phosphorus, and magnesium as the dominant elements within the crystals.


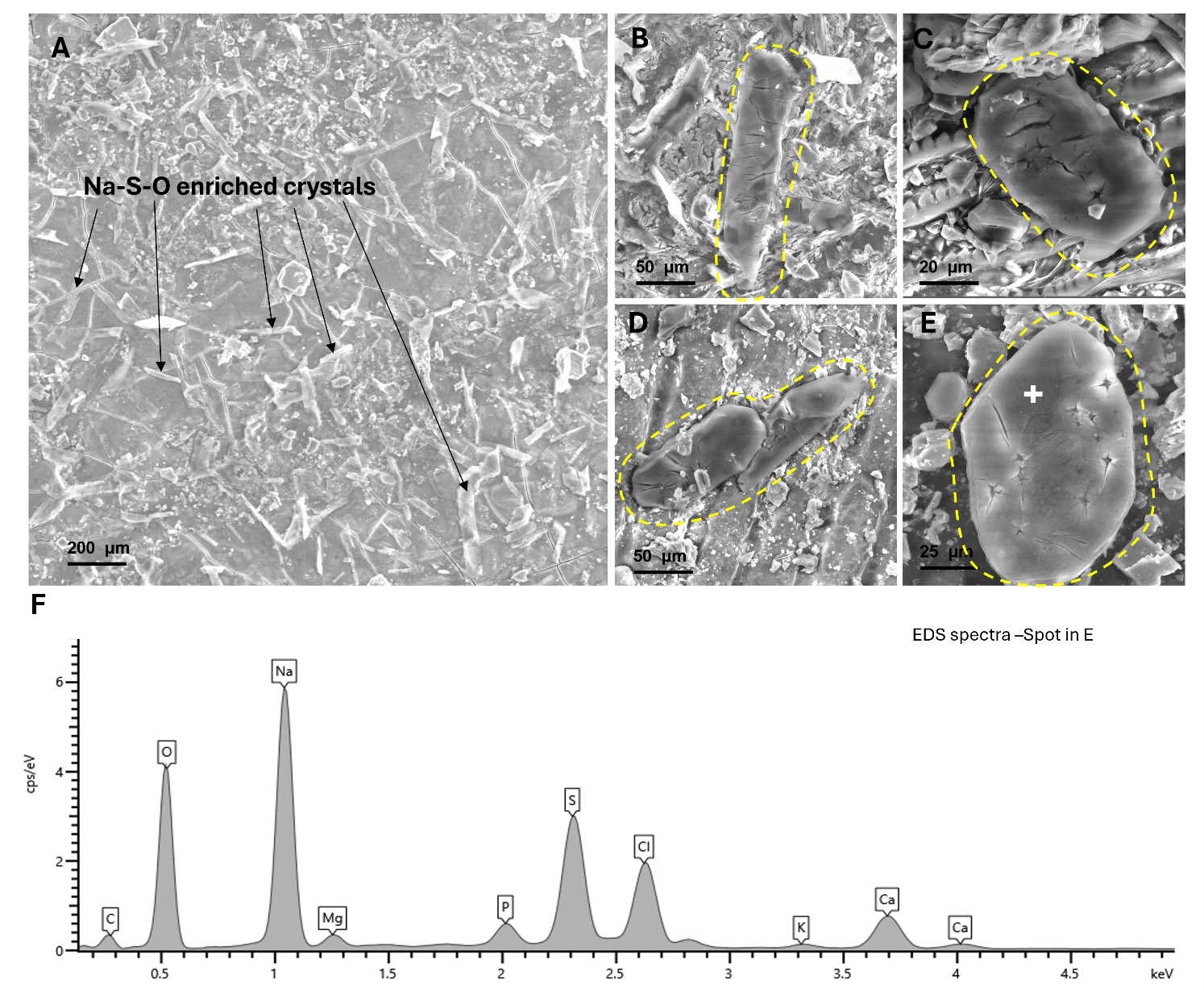


**Figure S8**. SEM images and EDS analysis of flat, irregular, smooth crystals observed throughout the incubation period. (A) Overview of mineral coverage on fish samples on day 16, showing these crystals as the predominant ones. (B) Mineral observed on day 8 under aerobic conditions. (C) Mineral observed on day 8 under anaerobic conditions. (D) Mineral observed on day 4 under aerobic conditions. (E) Mineral observed on day 4 under anaerobic conditions. (F) EDS analysis indicating that the main elements comprising these crystals are O, Na, S, and Cl, suggesting a sodium sulphate composition.


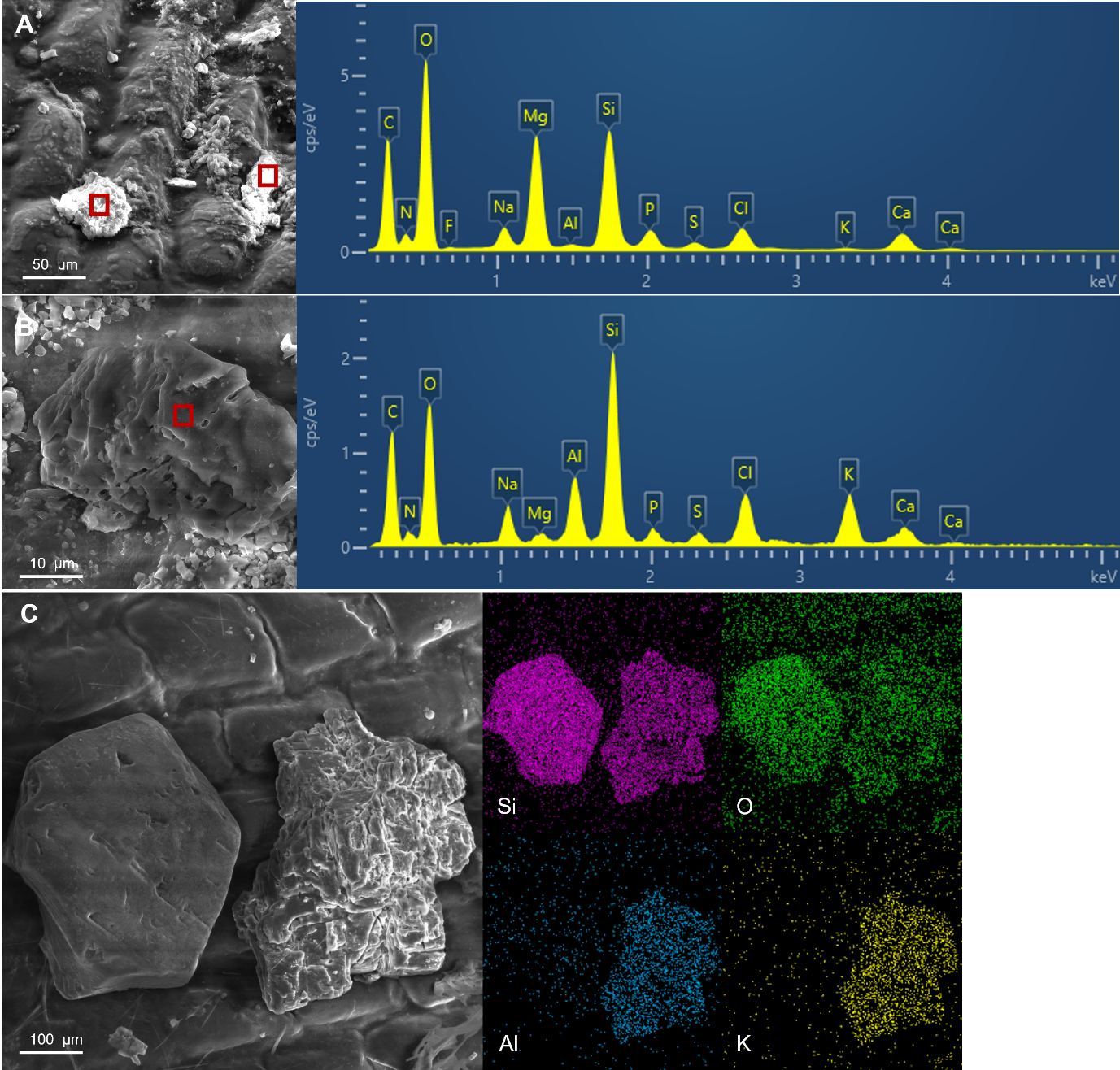


**Figure S9**. SEM images and corresponding EDS analysis of minerals observed on fish samples during the experimental period. (A) SEM image showing irregular crystals on the fish scales (day 4); the selected area yields an EDS spectrum dominated by O and Si, consistent with a silicate phase. (B) High-magnification SEM image of an irregular crystal (day 2) with an EDS spectrum similarly enriched in O and Si, supporting a silicate composition. (C) Low-magnification SEM image displaying two large irregular crystals with contrasting surface textures (day 2). EDS mapping reveals overlapping Si, Al, K, and O signals in one crystal, indicative of an aluminosilicate, while the other exhibits only Si and O, consistent with a silicate phase.


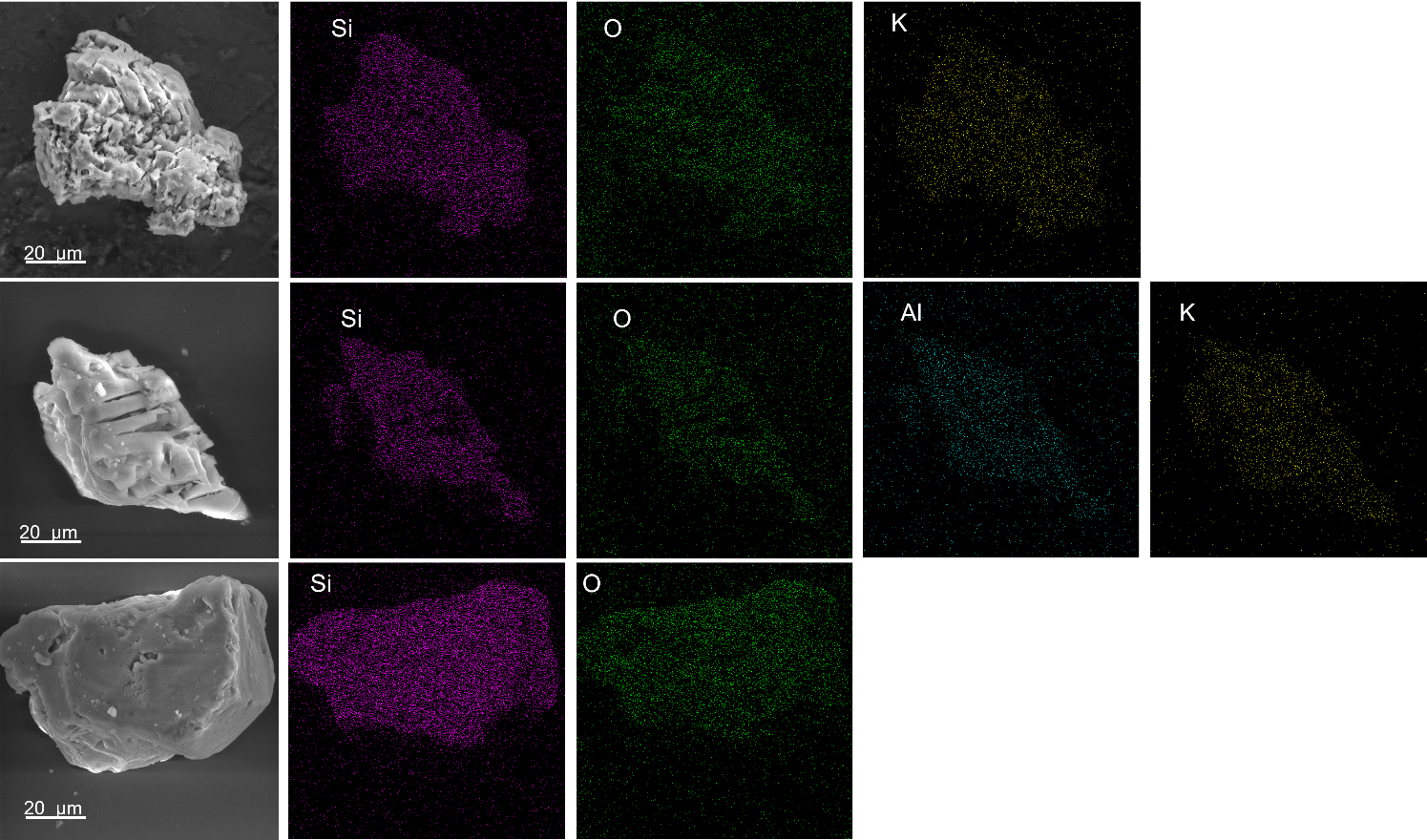


**Figure S10**. SEM images and corresponding EDS analysis of the raw sand (Westbuild, Australia) before use in the experimental setup for creating the mesocosms.


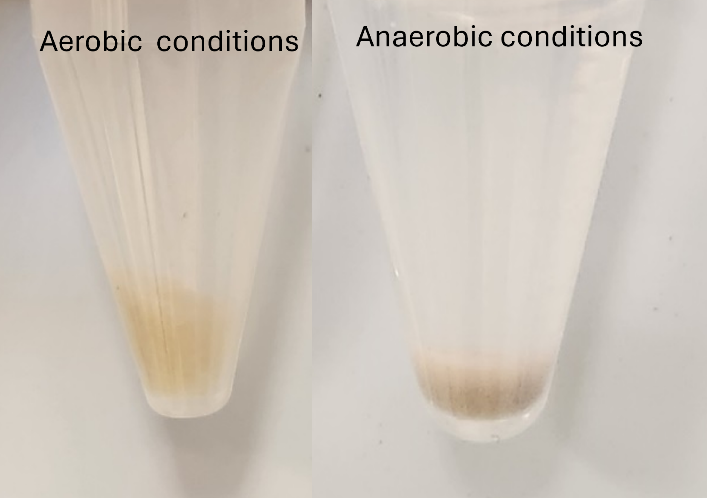


**Figure S11**. Photograph of mineral crust recovered from the water-air interphase at the end of the 16-day experiment.


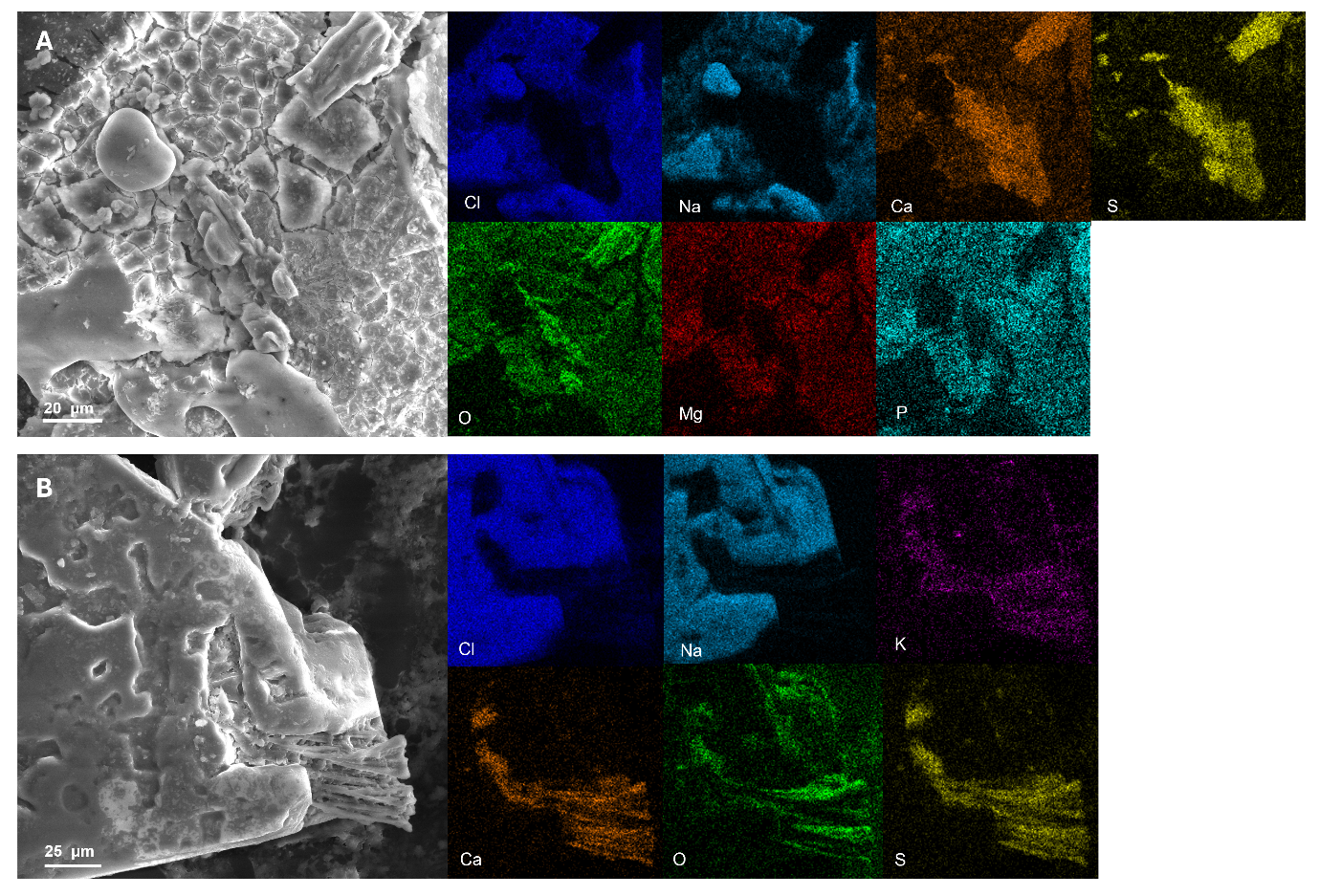


**Figure S12.** SEM images and EDS elemental maps of secondary crystals within the mineral crust precipitated at the air–water interface of experimental containers. (A) Aerobic condition: irregular Ca–S–O–rich crystals associated with halite, overlying the magnesium phosphate crust. (B) Anaerobic condition: bundles of needle-like Ca–S–O–rich crystals closely associated with a bigger halite crystal.





**Figure S13**. XRD diffractograms of the mineral crust formed at the air–water interface of experimental vessels under aerobic and anaerobic conditions, identifying struvite as the dominant phase.
